# Health status of honey bee colonies (*Apis mellifera*) and disease-related risk factors for colony losses in Austria

**DOI:** 10.1371/journal.pone.0219293

**Published:** 2019-07-09

**Authors:** Linde Morawetz, Hemma Köglberger, Antonia Griesbacher, Irmgard Derakhshifar, Karl Crailsheim, Robert Brodschneider, Rudolf Moosbeckhofer

**Affiliations:** 1 Department for Apiculture and Bee Protection, Institute for Seed and Propagating Material, Phytosanitary Service and Apiculture, Division for Food Security, Austrian Agency for Health and Food Safety Ltd., Vienna, Vienna, Austria; 2 Department for Statistics and Analytical Epidemiology, Division for Data, Statistics & Risk Assessment, Austrian Agency for Health and Food Safety Ltd., Graz, Styria, Austria; 3 Institute of Biology, University of Graz, Graz, Styria, Austria; Facultad de Veterinaria, Universidad de Murcia, SPAIN

## Abstract

Austrian beekeepers frequently suffered severe colony losses during the last decade similar to trends all over Europe. This first surveillance study aimed to describe the health status of Austrian bee colonies and to analyze the reasons for losses for both the summer and winter season in Austria. In this study 189 apiaries all over Austria were selected using a stratified random sampling approach and inspected three times between July 2015 and spring 2016 by trained bee inspectors. The inspectors made interviews with the beekeepers about their beekeeping practice and the history of the involved colonies. They inspected a total of 1596 colonies for symptoms of nine bee pests and diseases (four of them notifiable diseases) and took bee samples for varroa mite infestation analysis.

The most frequently detected diseases were three brood diseases: Varroosis, Chalkbrood and Sacbrood. The notifiable bee pests *Aethina tumida* and *Tropilaelaps* spp. were not detected. During the study period 10.8% of the 1596 observed colonies died. Winter proved to be the most critical season, in which 75% of the reported colony losses happened. Risks for suffering summer losses increased significantly, when colonies were weak in July, had queen problems or a high varroa mite infestation level on bees in July. Risks for suffering winter losses increased significantly, when the colonies had a high varroa mite infestation level on bees in September, were weak in September, had a queen older than one year or the beekeeper had few years of beekeeping experience. However, the effect of a high varroa mite infestation level in September had by far the greatest potential to raise the winter losses compared to the other significant factors.

## Introduction

The honey bee, important pollinator and producer of hive products, is threatened by a variety of pests and pathogens. Colony losses of managed honey bee colonies have been reported during winter but also summer from many countries [1–4]. Monitoring and surveillance studies have proven to be useful tools to address the problem of colony losses. Firstly, they describe the status-quo of bee health and can show trends in loss rates, if regularly conducted. Secondly, they guide towards improvements of bee health by hinting towards important factors through modelling. As disease occurrence and colony losses vary widely between different countries and climatic regions [1, 5–7], a complete picture of the distribution of bee diseases is necessary to understand problems on national but also on international level.

In Austria, systematic studies on winter mortality of managed honey bee colonies date back to 2007/08 [8]. In this publication, winter loss rates from 6.0% to 17.8% were reported for different Austrian regions. These surveys have been continued yearly and demonstrate a high fluctuation in the amount of winter losses between the years (minimum: 8.1%; maximum: 28.4%; [9, 10]) and between the regions within one year [11]. Additionally, they revealed several correlations between beekeeping practices and the amount of winter losses [1, 2, 8, 9, 12]. Generally, such surveys on winter losses are conducted by using self-administered questionnaires. Therefore they have to use clear and simple questions and are constricted to topics of common beekeeping knowledge to avoid misunderstandings and allow every interested beekeeper to participate in the survey [13]. Thus, the output from these surveys cannot and do not want to replace the data drawn from professional colony inspections conducted by an expert. Comparing the results of these two experimental designs can give valuable insights into the validity of the collected data as well as the strengths and weaknesses of the respective methods.

The health status of Austrian bee colonies was surveyed during several projects in the recent years conducted by AGES (Austrian Agency of Health and Food Safety) [14–16]. In these projects colony inspections were conducted by trained AGES staff and samples were analyzed in the lab for different pests and bee diseases. However, all studies were designed as passive monitoring and relied on beekeepers to report problems with their colonies. Therefore their results were not representative for Austrian beekeepers.

In contrast, the data of the present active surveillance study enables us to make verified statements about the disease prevalence and the health status of Austrian colonies for the first time. The study was conducted by trained bee inspectors, who collected information from 189 randomly selected apiaries all over Austria by questionnaire, colony inspections and sampling. Hence, we provide these data from Austria as another important puzzle piece towards completing the disease landscape in bees. They are especially valuable, as Austria did not participate in the European-wide EPILOBEE project [5, 17], which surveyed the bee health in the years 2012 to 2014.

The aim of the presented work was to evaluate the health of Austrian honey bee colonies and to describe risk factors for their loss connected with bee diseases and beekeeping practice. Thus, the prevalence of infection and disease of pathogens and parasites in Austria was determined at three times of the year (summer, autumn, spring). Furthermore, factors related to beekeeper characteristics, colony status and health were analyzed in correlation with colony losses in summer and in winter. We defined the main problems in bee health especially for Austria and ranked the risk factors for each season and the strength of their impact.

## Materials and methods

### Apiary selection

**Fig 1:**
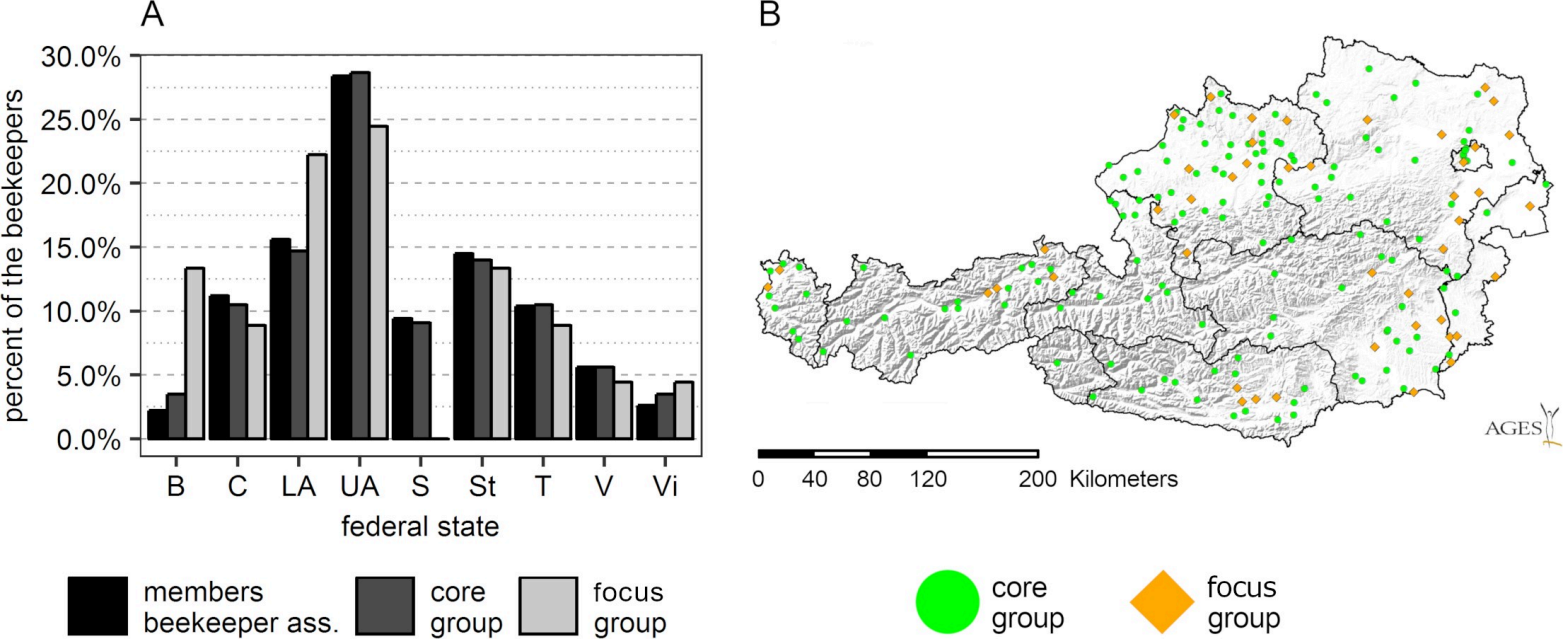
Distribution of the participating beekeepers in the nine federal states of Austria. (A) Comparison of the amount of beekeepers in the different federal states (25 207 beekeepers registered in the Austrian Beekeepers Association for the year 2013, black bars), the amount of participants in the core group (144 beekeepers, dark gray bars) and the focus group (45 beekeepers, light gray bars). (B) Map of distribution of all 189 participating beekeepers (green circle: core group, orange diamond: focus group). B = Burgenland, C = Carinthia, LA = Lower Austria, S = Salzburg, St = Styria, T = Tyrol, UA = Upper Austria, Vi = Vienna, V = Vorarlberg.

In spring 2015, 200 apiaries from all over Austria were selected for participation in the surveillance study (one apiary per beekeeper, for details see S1 Text). A total of 189 beekeepers remained in the project for the whole timespan (summer 2015 to spring 2016) and were therefore included in the analysis (Fig 1, S1 Dataset). However, calculation of winter losses was based on 188 apiaries as one apiary lost all colonies before winter. The sample of the participating beekeepers consisted of two subgroups: (1) a core group, which represented the distribution of beekeepers in Austria (Fig 1A; N = 144) and (2) a focus group, which comprised beekeepers of special interest (N = 45). The latter group was created to increase the amount of apiaries in the sample with the following attributes: from areas with high winter losses during the preceding winter, from areas with frequent suspected bee poisoning incidents, from urban areas and maintained by professional beekeepers. The apiaries of the focus group were located mainly in the East of Austria, as most professional beekeepers and the main agricultural production areas are located in this part of Austria (Fig 1B). Apiaries from urban areas were included into the focus group so that this presently emerging type of urban beekeepers (few colonies, beginners, urban environment) is also represented in the sample.

### Field inspections

Data collection of this study was conducted from summer 2015 to spring 2016. All participants were visited three times by bee inspectors (for definition see below): the first visit was conducted in summer 2015 before the start of the main treatment against the varroa mite (91% of the visits in July, 9% in August). The second visit was conducted in autumn 2015 after the end of the main treatment against the varroa mite (85% of the visits in September, 15% in October). The third visit was conducted in spring 2016 after overwintering (88% of the visits in March/April, 12% of the visits in May/June). The wide range of visit dates was due to climatic differences between the regions (e.g. late spring visits of apiaries located at higher altitudes) and due to coordination arrangements between bee inspectors and beekeepers. No specific sampling permissions were required for conducting the inspections, because *Apis mellifera* is not listed as endangered or protected species in Austria and the participating beekeepers gave their permission for the inspections and sampling activities.

The visits were conducted by experienced bee inspectors, who were specially trained in a one-day class within the project. During each visit, the bee inspectors filled in a questionnaire about the characteristics of the beekeeper, the beekeeping operation, the visited apiary and about the conditions of each inspected colony (S2 and S3 Text). Colony inspections were conducted by estimating the colony size and examining the bees, the brood combs and the front of the colony for signs of pests and diseases. Bee inspectors either diagnosed a disease on-site or took samples of suspicious material for laboratory analysis (details see Table 1). In summer and autumn, they took also bee samples for the analysis of the varroa mite infestation level.

**Table 1:** Summary of the clinical signs used to detect the particular diseases during the colony inspections.

Disease	Clinical symptoms	Sample matrix	References
American Foulbrood (AFB)	patchy brood pattern, cell cappings concave, punctured or discolored, glue-like larval remains (shown by the match stick test for ropiness), typical AFB smell, tightly adherent scales	brood sample with symptoms	[18–20]
Chalkbrood	chalkbrood mummies (white or gray) loosely in brood cells*, chalkbrood mummies on the bottom board*, patchy brood pattern	no sample, on-site diagnosis	[21, 22]
Chronic Bee Paralysis Virus (CBPV)	black and shiny bees, loss of hair, trembling motion of wings and body, rejected bees and crowded entrance, bees are unable to fly and crawl on the ground in front of the hive, signs of diarrhea	10 bees with symptoms	[23, 24]
European Foulbrood (EFB)	patchy brood pattern, cell cappings punctured, color of larvae turns to yellow and brown, larvae are displaced inside the cell, slumped larvae, loose scales, discolored dead larvae in open brood cells, sour or foul smell	brood sample with symptoms	[22, 25]
Nosemosis	dead or flightless bees in front of the hive, fecal marks, bees with dilated abdomens, depopulation	30 bees with symptoms	[26]
Sacbrood	dead larvae with saclike appearance and fluid under larval skin*, ‘gondola-shaped’ scales*, patchy brood pattern, cell cappings punctured	no sample, on-site diagnosis	[24, 27]
Varroosis	varroa mites on adult bees*, bees with deformed wings*, bees with deformed abdomens, patchy brood pattern, brood cell cappings punctured, discolored larvae/pupae, dead larvae/pupae, varroa mites embedded in wax cappings of brood cells*	no sample, on-site diagnosis	[22, 24]

* Typical symptoms for identifying the bee diseases Chalkbrood, Sacbrood and Varroosis, which were identified directly in the apiary without any lab analysis.

In cases of suspicion for a certain disease, bee or brood samples were taken for laboratory analysis. This table does not present all of the symptoms of the given diseases exhaustively but shows the list of symptoms the bee inspectors were advised to look for (see S2 and S3 Text).

In apiaries with more than ten colonies, ten colonies were selected randomly for the surveillance study (= study colonies). If ten or less than ten colonies were present in the apiary, all colonies were sampled. Bee inspectors noted if the colonies were newly established. The strength of each bee colony was approximately estimated by the bee inspector using three categories: (1) more or less normal/average sized colony, (2) very small/weak colony, (3) very big/strong colony. The bee inspectors were asked to take into account the colony size typical for each particular region and time of the year. A colony was defined as dead, when it did not contain any living bees. However, in five cases bee inspectors defined colonies as dead, although a small amount of bees was still alive and they took bee samples. In all five cases additional information provided by the bee inspectors made it evident, that these colonies were not able to survive. Therefore we maintained the classification of these colonies as ‘dead’. Queen failure was diagnosed by the bee inspector, when queen problems were obvious at the time of inspection such as: no queen at all, no egg-laying queen, egg-laying workers or drone-laying queen.

Bee inspectors recorded if clinical signs of American Foulbrood (AFB), Chronic Bee Paralysis Virus (CBPV), Chalkbrood, European Foulbrood (EFB), Nosemosis, Varroosis or Sacbrood were visible (Table 1 for list of clinical signs). Furthermore, they searched visually for the bee pests *Aethina tumida* (Small Hive Beetle) [28, 29] and *Tropilaelaps* spp. (Tropilaelaps-mite) [30]. Samples with suspicion for AFB (brood sample), *A*. *tumida* (samples of the suspicious beetle, larvae or eggs) or Tropilaelaps mites (samples of suspicious mites) had to be sent to AGES for confirmation or further investigation, as these are notifiable diseases [31]. Positive cases of AFB were notified to the authorities. The occurrence of the parasitic disease Varroosis had not been notified, because it is only a notifiable disease in Austria when occuring in epidemic extend.

The brood diseases Chalkbrood, Varroosis and Sacbrood were identified directly in the apiary by the bee inspectors by the typical clinical signs (Table 1). In cases of clinical signs of Nosemosis or CBPV, adult worker bees with clinical signs were sampled and sent to the AGES-lab for diagnostic clarification.

Additionally, an analysis for varroa mite infestation level was conducted at the summer visit and the autumn visit. Thus, the infestation status of a study colony by the varroa mite was determined with the help of two measurements: (1) the colony inspection, during which the colony was checked for Varroosis (for details see Table 1) and (2) the determination of the infestation level of adult worker bees by varroa mites (varroa mite infestation level). Please note that for calculating the prevalence of Varroosis, we took only into account the data about clinical signs of Varroosis (Table 1) and not the data about varroa mite infestation level on bees. In 56 cases no bee sample was taken due to the small size of the colony (summer: 33 colonies, autumn: 23 colonies). Thus, these samples were missing in a systematic way and the missing data could theoretically distort the modelling. We tested for such distorting effects on the modelling, but found no evidence (for details see S4 Text).

### Laboratory tests

#### American foulbrood

Bee colonies were suspicious for AFB, when clinical signs of the disease were found during inspection (Table 1). A brood sample was taken and tested for the presence of the etiological agent *Paenibacillus larvae* in the laboratory. The culture method in the laboratory was carried out according to the internal standard operating procedure based on the OIE terrestrial manual [20]. In a first step, material from the diseased brood was inoculated onto Columbia sheep blood agar and Columbia sheep blood slant agar. Suspicious bacteria colonies were identified visually by morphological characteristics and tested for their catalase reaction [32]. Additionally, the liquid phase from the inoculated slant agar tubes was checked by light microscope (magnification 400-1000x) for giant whips, which are formed during the sporulation process by *P*. *larvae* [33, 34].

#### Nosemosis

Bee samples with symptoms of Nosemosis (Table 1) were tested in a first step for the presence of *Nosema* spp. spores. A homogenate was produced by grinding 30 bee-abdomina with 5 ml water according to the internal standard operating procedure [35]. The suspension was analyzed microscopically (magnification 200-400x) for *Nosema* spp. spores. The positive samples were further analyzed to separate between the species *N*. *apis* and *N*. *ceranae*. For this DNA extraction was performed employing the ‘High Pure PCR Template Preparation Kit‘ (Roche) [36, 37]. DNA was extracted from 2 ml homogenate, which had been diluted to 1 ml per bee. PCR was conducted according to a multiplex PCR-protocol using ‘REDTaq Readymix PCR Reaction Mix’ (Sigma) ([38], for primers see S1 Table). The PCR-parameters were: 94°C 2 min; 10 cycles: 94°C 15 s; 61,8°C 30 s; 72°C 45 s; 20 cycles: 94°C 15 s; 61,8°C 50 s; 72°C 50 s; followed by an elongation step at 72°C for 7 min [38].

#### CBPV

Bee samples with symptoms of a CBPV infection (Table 1) were molecularly analyzed. The samples of 10 pooled bees were homogenized in 3 ml DEPC treated water in blender bags with a homogenizer (Bioreba). RNA-extraction was done employing “QIAamp viral RNA Mini Kit” (Qiagen) [14]. Viral RNA was reverse-transcribed and amplified in a one-step RT-PCR-method by using the ‘OneStep RT-PCR Kit’ (Qiagen). The PCR-parameters were: 50°C 30 min; 95°C 15 min; 30 cycles: 94°C 30 s; 57°C, 30 s; 72°C 45 s followed by an elongation step at 72°C for 10 min ([39], for primers see S1 Table).

The CBPV symptom ‘trembling motion of wings and body’ was described to be elicited also by the other bee viruses Acute Bee Paralysis Virus and Slow Bee Paralysis Virus after experimental infection [40, 41]. Thus, we cannot exclude, that bees tested negatively for CBPV were suffering from another virus disease.

#### Estimation of varroa mite infestation level of adult bees

During colony inspection a 125 ml cup full of bees was collected from a brood comb (mean ± s.d.: 316 ± 71 bees per sample, s.d. = standard deviation). Each sample was weighed to be able to calculate the total number of bees per sample in a second step. Then each complete sample was washed with 0.1% soapy water and the number of varroa mites per sample was counted [42]. The varroa mite infestation level was calculated as percentage for each sample (number of varroa mites *100 / number of bees). Please note, that a varroa mite infestation level of zero does not mean that the colony is free of varroa mites, but that the varroa mite infestation level is below the detection threshold for the soapy water method (= less than one varroa mite in the approximately 300 analyzed bees).

### Statistical analysis

All colonies with continuous records during the whole study or which died during the study were included into the dataset. Accordingly, we excluded colonies from the dataset for two reasons: they were removed from the apiary during the study (2 cases) or they were merged with other colonies (20 cases) and were therefore lost for the record. In the end, the dataset included 1596 colonies for summer losses and 1554 colonies for winter losses (difference in colony number between the two datasets: 42 colonies due to summer losses). Because of missing values of certain factors (varroa mite infestation level, queen age, etc.) the number of colonies decreased for the univariate and multivariate analysis accordingly (S2–S4 Tables).

Statistical analyses were conducted using R Version 3.4.1. [43]. Generally, the clinical prevalence is defined as the proportion of infected individuals by a particular pest or parasite [44]. The prevalence of the nine diseases was calculated on apiary level, i.e. it gives the proportion of infected apiaries instead of the number of infected individuals or colonies (as given in the prevalence definition above). Thus, an apiary was rated as positive if at least one colony showed clinical symptoms of the respective disease. For each disease a General Linear Model (GLM) with quasibinomial distribution was calculated [13] and 95% Confidence Intervals (95%CI) were extracted from the particular model using the command ‘confint’. Posthoc tests for prevalence differences between seasons were performed with Tukey Tests using the command ‘glht’ (package ‘multcomp’, [45]).

The probabilities of summer and winter losses were calculated with General Linear Mixed Models (GLMM) with binomial distribution and apiary identity as random factor (package `lme4’, [46]). A total of 29 variables describing beekeeper and colony characteristics were available for testing their influence on summer or winter colony loss (S2–S4 Tables). These were preselected in univariate analyses using Pearson’s Chi^2^-Test, Fisher Exact Test and Wilcoxon Rank Sum Test, respectively. All variables with a significant correlation with loss were used in building the particular GLMM in a stepwise approach with forward selection. Models were compared by ANOVA’s, additionally the Akaike information criterion (AIC) was considered. The selection process was stopped when the addition of a new variable did not improve the significance of the model or when Δ AIC was smaller than 2 [47]. Probability calculations and visualisations of the models were extracted with the package ‘sjPlot’ [48]. Graphs were drawn using the package ‘ggplot2’ [49] and ‘scales’ [50].

## Results

### Description of the participating beekeepers

The core group and the focus group did not differ in the frequency of the summer or winter losses in the project period 2015/16 (core group: 8.6% winter losses, 95%CI 7.1–10.3%, 2.7% summer losses, 95%CI 1.9–3.7%; focus group: 7.9% winter losses, 95%CI 5.5–10.8%, 2.4% summer losses, 95%CI 1.2-4.2%). Therefore, this factor was not included into the further analyses.

The surveillance study included the whole range of beekeepers, from small backyard beekeepers to professional beekeepers. Participating beekeepers owned a mean of 67 colonies (±134.6 s.d.). Two beekeepers owned a minimum of two colonies, whereas the largest beekeeping operation owned 1200 colonies. However, most participants (72%) owned less than 50 colonies.

Similarly, the colony numbers in the visited apiaries ranged from two colonies to 71 colonies. At mean, the surveillance apiaries consisted of 14.9 colonies (±9.3 s.d.). 37% of the apiaries consisted of 10 or less colonies, 42% of the apiaries consisted of 11 to 20 colonies and 21% of the apiaries consisted of more than 20 colonies. The size of the apiary correlated positively with the size of the beekeeping operation (Spearman’s rank correlation: r_S_ = 0.66).

Participating beekeepers had a mean of 24.8 years of beekeeping experience (±16.2 s.d.). Five beekeepers had started with beekeeping the year before the surveillance study. One beekeeper had been working with bees for 70 years at the start of the study. 88% of all participants had attended classes or trainings in beekeeping and 37% had completed a professional education in beekeeping (S3 Table, master beekeeper, skilled worker in beekeeping, etc.). The beekeeper’s years of experience correlated positively with size of the beekeeping operation (Spearman’s rank correlation: r_S_ = 0.39) although the correlation coefficient is low. 16.9% of all participants practiced organic beekeeping according to one of the organic certification labels of Austria (e.g. AMA-Biosiegel, Demeter).

### Health status of the bee colonies

At the first visit, two thirds of the inspected colonies were classified by the bee inspectors as normally sized colonies. 12% of the colonies were classified as very weak and 20% of the colonies were classified as very strong colonies (N = 1522 classified colonies 1^st^ visit, S3 Table). This pattern persisted during the following two visits (Pearson’s Chi^2^ Test: Chi^2^ = 2.23, df = 4, P = 0.694). Colonies rated as very weak showed at least one of the following colony characteristics in summer and/or in autumn with a significantly increased probability: queen failure, new queen introduced, colony newly established (nuc, swarm, etc.), clinical signs of AFB, clinical signs of Varroosis and clinical signs of Sacbrood (for details see S5 and S6 Tables).

Beekeepers were instructed to handle their colonies in their usual manner and treat them against the varroa mite according to their own concept. Therefore, all beekeepers treated their bees against the varroa mite in summer 2015 and winter 2016 –however, the methods and substances used varied broadly (formic acid, oxalic acid, thymol, brood removal, queen caging, etc.).

**Fig 2:**
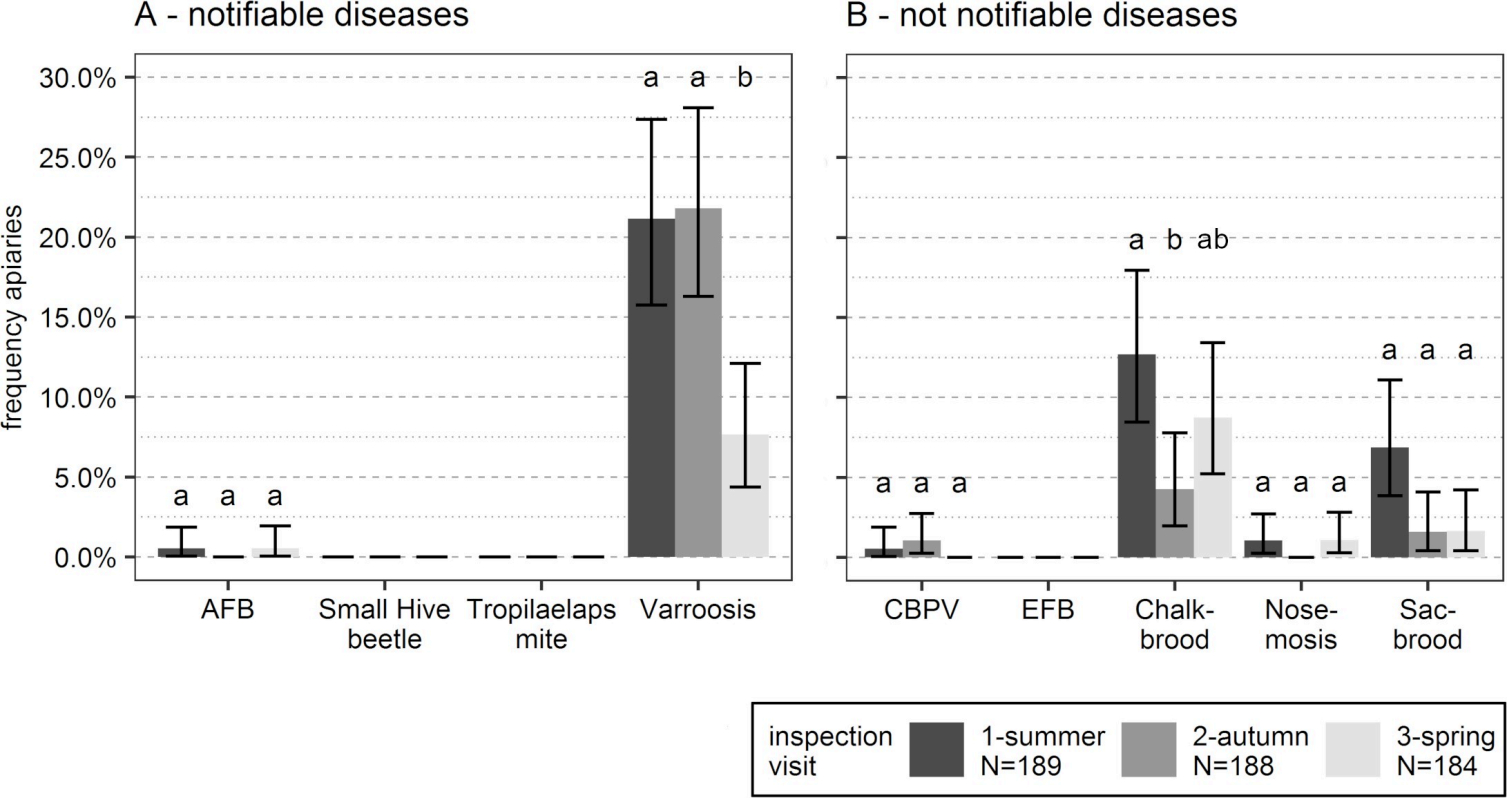
**Clinical prevalence of (A) notifiable and (B) not notifiable bee diseases in Austrian apiaries.** N = number of inspected apiaries. Differences in the prevalence between the seasons were tested for each detected disease separately, different letters report significant differences between the seasons (posthoc Tukey-test: P<0.05). Error bar: 95%CI.

Clinical cases of AFB, Varroosis, CBPV, Chalkbrood, Nosemosis and Sacbrood were observed and reported by the bee inspectors during the study period (Fig 2, S7 Table). Neither the notifiable bee pests *A*. *tumida* and *Tropilaelaps* spp., nor the disease EFB were detected (Fig 2).

The parasitic disease Varroosis was most frequently observed; 186 clinical observations were reported during the three visits. In summer and autumn, clinical prevalence of Varroosis was reported for approximately 21% of the apiaries (Fig 2A, S7 Table). Varroosis was three times more frequent in summer and autumn than in spring (Tukey posthoc test: P < 0.01 for both comparisons). In total, 166 colonies from 73 apiaries showed symptoms of Varroosis. In 20 of these colonies, Varroosis was detected at two different visits. The following symptoms were observed during inspections, when Varroosis was diagnosed: varroa mites on adult bees (118 observations), bees with deformed wings (88 observations) and varroa mites embedded in wax cappings of brood cells (23 observations).

AFB was reported for three colonies during the summer visit and one colony during the spring visit. The clinical prevalence was always below 1% of the apiaries. Prevalence did not differ between the visits (Fig 2A, S7 Table, Tukey posthoc test: P > 0.05 for all comparisons). The three positive cases in summer were all detected in one apiary. Subsequently, all colonies of that apiary were culled because of AFB.

Chalkbrood was observed in 100 colony inspections during the course of the three visits. Clinical prevalence was between 8.7% and 12.7% of the apiaries (Fig 2B, S7 Table). It was detected significantly more often in colonies in summer than in autumn (Tukey posthoc test: P < 0.05). In total, 77 colonies in 29 apiaries showed clinical Chalkbrood, of which 15 colonies were positive for Chalkbrood symptoms at two visits and four colonies at all three visits.

CBPV was reported and verified by lab analyses for three colonies from three different apiaries. Clinical prevalence ranged between 0.0% and 1.1% of the apiaries (Fig 2B, S7 Table) and did not differ between the three visits (Tukey posthoc test: P > 0.05 for all comparisons). In all three colonies the symptom ‘black and shiny bees’ was reported, in one of the colonies the symptom ‘faecal marks’ was additionally observed.

Nosemosis was reported in eight colonies from four different apiaries and verified by lab analyses. Its clinical prevalence ranged from 0.0% in autumn to 1.1% in summer and spring (Fig 2B, S7 Table). It did not differ significantly between the three visits (Tukey posthoc test: P > 0.05 for all comparisons). Bee inspectors reported dead bees in front of the colony (five cases), signs of diarrhea (one case), dead bees in front of the colony together with signs of diarrhea (one case) and flightless bees in front of the colony (one case). The analyzed matrix was ‘dead bees’ in seven cases, and ‘sampled feces’ in one case. With the seven bee samples, additional analyses to identify the *Nosema* species were conducted. Three samples were infected with *N*. *ceranae*, three samples were infected with *N*. *apis*, and one sample revealed coinfection with both species.

Sacbrood was reported in 26 colony inspections in the study period, whereas 77% of the cases were detected during the summer visit (Fig 2B, S7 Table). The prevalence varied between 1% and 7% of the apiaries, dependent on the visit season. It did not differ between the summer visit and the two other visits (Tukey posthoc test: P ~ 0.06 for both comparisons). In all colonies positive for Sacbrood, the symptoms were found just at one visit.

**Fig 3:**
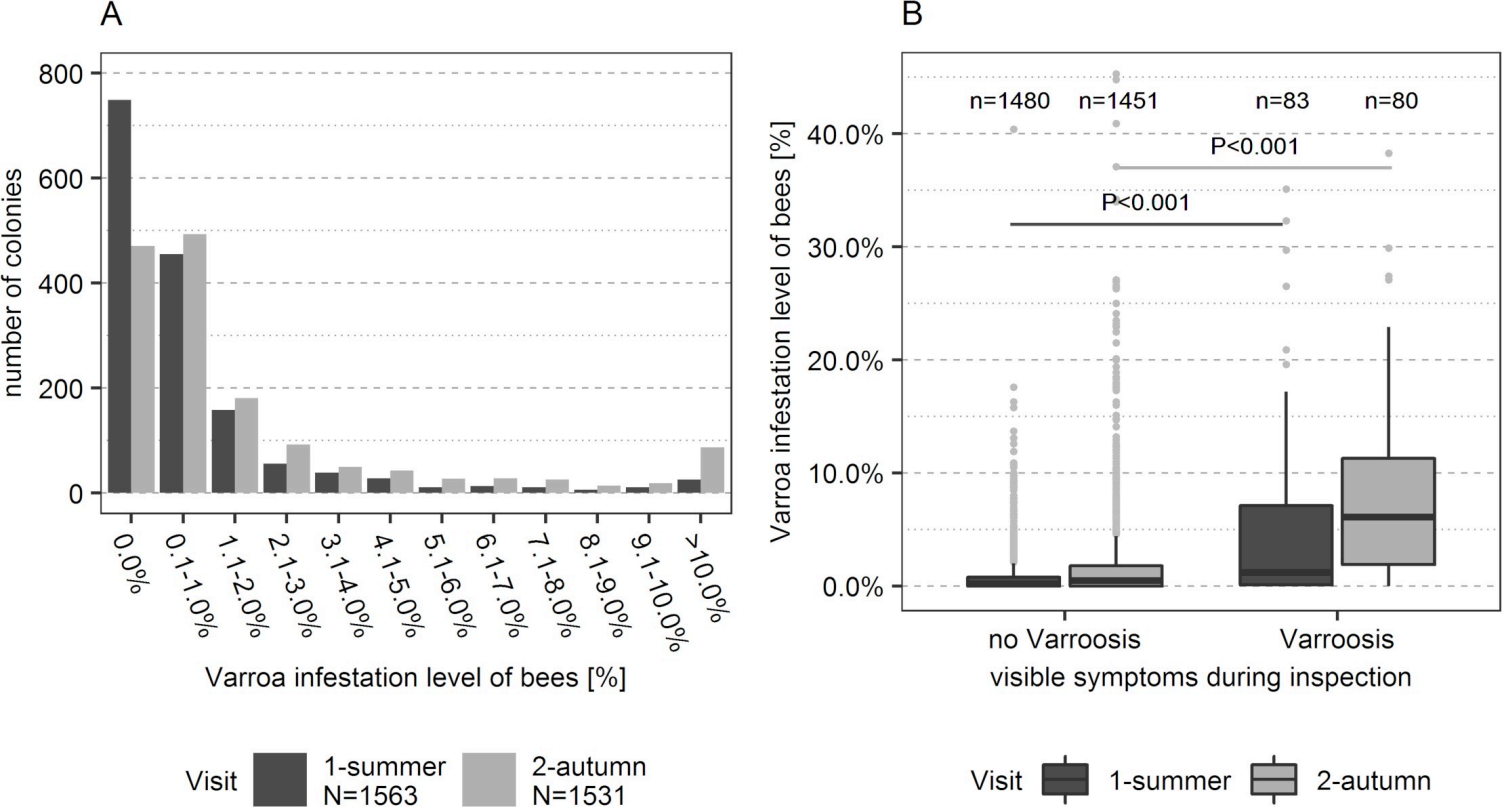
Varroa mite infestation level and Varroosis. (A) Varroa mite infestation level of bees in the monitored bee colonies from 189 (1^st^ visit, summer 2015) and 188 apiaries (2^nd^ visit, autumn 2015), respectively. Maximum measured infestation levels were 40.4% (1^st^ visit) and 137.9% (2^nd^ visit) respectively. (B) Relation between varroa mite infestation levels on bees and symptoms of Varroosis in the respective bee colonies separated for sampling event. Four outliers of the second visit between 40% and 138% are not shown on the graph. Statistics: Wilcoxon rank sum test.

Additionally, bee samples were taken in summer and autumn to determine the varroa mite infestation level of the monitored colonies. In summer, varroa mites were detected in 52% of the 1563 bee samples (Fig 3A). The median varroa mite infestation level was 0.3% (first quartile-third quartile (Q1-Q3): 0.0-0.9%; maximum: 40.4%). In autumn, varroa mites were detected in 68% of the 1531 bee samples (Fig 3A). The median varroa mite infestation level was 0.6% (Q1-Q3: 0.0-2.1%; maximum: 137.9%). The maximum value of 137.9% was measured in an apiary with heavy varroa infestation problems (median varroa infestation level in autumn: 19.8%, Q1-Q3: 5.1-34.0%). At the time of sampling, half of the colonies were already dead and all other colonies except one died in the subsequent months–very likely from Varroosis. A sample without varroa mites signifies that the infestation level was under the detection threshold of the soapy water method (i.e. no varroa mite in the sample), but not that the particular colony was free of varroa mites.

The analysis of the varroa mite infestation level of the bees confirmed the diagnoses by the bee inspectors concerning Varroosis (Fig 3B). Varroa mites were found significantly more often in colonies diagnosed positive for Varroosis than in the other colonies (Wilcoxon rank sum test: summer: W = 35870, P < 0.001; autumn: W = 22362, P < 0.001). Varroa mites were found in 83% of all bee samples from colonies diagnosed with Varroosis and in 59% of all samples of colonies without Varroosis. During both, the summer and the autumn visit, colonies diagnosed with Varroosis had a six times higher varroa mite infestation level than colonies without Varroosis (Fig 3B). At the summer visit, colonies without Varroosis had a median varroa mite infestation level of 0.2% (Q1-Q3: 0.0-0.8%, n = 1480 colonies) and colonies diagnosed with Varroosis had a median varroa mite infestation level of 1.2% (Q1-Q3: 0.1-7.1%, n = 83 colonies). At the autumn visit, colonies without Varroosis had a median varroa mite infestation level of 0.5% (Q1-Q3: 0.0-1.8%, n = 1451 colonies) and colonies diagnosed with Varroosis had a median varroa mite infestation level of 6.1% (Q1-Q3: 1.9-11.3%, n = 80 colonies).

### Colony losses

Colony losses were generally low throughout the study. Three quarters of the colony losses were recorded in the winter season. In sum 173 of the 1596 observed colonies died between summer 2015 and spring 2016 (10.8%). 42 colonies from 22 apiaries died in summer between the first and second visit (2.6% colony loss, 95%CI 1.9-3.5%). In one apiary, all nine colonies were culled because of AFB and therefore the number of inspected apiaries was reduced from 189 to 188. In autumn, 1554 colonies were alive, of which 131 colonies died during the following winter season (8.4% colony loss, 95%CI 7.1-9.9%). Winter loss of study colonies was recorded in one third of the apiaries. In apiaries with winter losses, a mean of 29% (± 21% s.d.) of all study colonies died. The maximum winter loss of study colonies per apiary was 80% (3 cases). In 127 apiaries, all study colonies survived the winter 2015/16 (67.6% of all apiaries).

### Univariate analyses for variable selection

Before model building, univariate analyses were conducted to decide which variables to use for the modelling. 29 variables connected with colony health, colony characteristics and beekeeper characteristics were tested for correlation with summer and winter loss, respectively (Table 2, S2–S4 Tables for details and test statistics). Seven variables showed a significant correlation with summer loss and eight variables with winter loss. These were used as basis to build the two multivariate models.

**Table 2:** Summary of the univariate testing for the risk factors of summer and winter losses, respectively.

Data level	Factor	Possibility of summer losses	Possibility of winter losses
**Diseases summer visit**	AFB	**increases**	no effect
	CBPV	no effect	no effect
	Chalkbrood	no effect	no effect
	Nosemosis	no effect	no effect
	Sacbrood	no effect	no effect
	Varroosis	**increases**	**increases**
	High varroa mite infestation level	**increases**	**increases**
**Diseases autumn visit**	AFB	—	no effect
	CBPV	—	no effect
	Chalkbrood	—	no effect
	Nosemosis	—	no effect
	Sacbrood	—	no effect
	Varroosis	—	**increases**
	High varroa mite infestation level	—	**increases**
**Colony characteristics**	Nuc colony in spring	no effect	**decreases**
	Colony migrated	no effect	no effect
	Colony weak in summer	**increases**	no effect
	Colony weak in autumn	—	**increases**
	Old queen in summer	no effect	—
	Old queen in autumn	—	**increases**
	Queen failure in summer	**increases**	—-
	Queen failure in autumn	—	no effect
	Type winter food	—	no effect
	Amount of honey harvest	—	no effect
**Beekeeper characteristics**	Large beekeeping operation	**decreases**	no effect
	Large apiary	**decreases**	no effect
	Level of beekeeping education	no effect	no effect
	Many years of experience	no effect	**decreases**
	Organic beekeeping	no effect	no effect

‘increases’ = significant increase (P<0.05) of the probability of colony loss, when factor was applicable to the colony; ‘decreases’ = significant decrease (P<0.05) of the probability of colony loss, when factor was applicable to the colony; ‘no effect’ = no significant effect (P>0.05) on the probability of colony loss; ‘—’ = not tested. Descriptive statistics and test statistics can be found in the supplements (S2–S4 Tables).

The possibility of summer loss was positively correlated with symptoms of Varroosis in summer, symptoms of AFB in summer, high varroa mite infestation level on adult bees in summer, queen failure in summer and a weak colony size in summer (Table 2). It was negatively correlated with large beekeeping companies and large apiaries. The following factors were not used for model building, as there was no significant influence on summer losses: symptoms of CBPV, Chalkbrood, Nosemosis or Sacbrood; colony type, migration, old queens, beekeeping education, beekeeping experience and organic beekeeping.

The possibility of winter loss was positively correlated with symptoms of Varroosis in summer and autumn, high varroa mite infestation level on adult bees in summer and autumn, a small colony size in autumn and old queens in autumn (Table 2). It was negatively correlated with years of beekeeping experience and with colonies started as nucs in spring. The following factors were not used for model building, as there was no significant influence on winter losses: symptoms of AFB, CBPV, Chalkbrood, Nosemosis or Sacbrood; migration, colony size in summer, queen failure in autumn, type of winter food, amount of honey harvest, size of beekeeping operation, size of apiary, level of beekeeping education and organic beekeeping. However, clinical signs of the diseases Nosemosis and Sacbrood appeared more often in colonies, which died during winter, than in the surviving colonies (S2 Table). These diseases were very seldom and the test result may be falsely non-significant because of the low sample size.

### Summer losses: Multivariate analysis

The final GLMM included the variables varroa mite infestation level in summer, colony size in summer and queen failure in summer (Table 3). The following variables were not included in the final model, because the multivariate analysis showed no significant correlation with summer loss: operation size, apiary size, signs of Varroosis and signs of AFB. Signs of AFB were rare (three colonies in one apiary) and therefore the variable had no effect in the model. However, all colonies in the dataset with AFB-symptoms died.

**Table 3:** Explanatory factors for summer losses in Austria between July 2015 and September 2015.

Variable	Levels	Estimate (SE)	Odds (95% CI)	Z value	P
Intercept		-11.15 (1.79)	0.00 (0.00–0.00)	-6.23	**< 0.0001**
Mite infestation level summer		0.15 (0.08)	1.17 (1.00–1.35)	1.88	**0.0468**
Colony strength summer reference level: strong	Normal	1.53 (1.01)	4.63 (0.63–33.77)	1.51	0.1309
	Weak	3.49 (1.19)	32.77 (3.21–334.74)	2.93	**0.0033**
Queen failure reference level: queenright	Queenless	4.15 (1.51)	63.37 (3.31–1211.35)	2.75	**0.0059**

GLMM with binomial distribution, random factor ‘apiary’. The random factor improved the model significantly (Chi^2^ = 89.93, df = 1, P < 0.001). n = 1399 colonies; N = 189 apiaries, SE = standard error.

A typical summer loss colony was queenless, weak or highly infested with varroa mites when inspected in July (Table 3). Summer losses were generally rare; the model contained 38 cases of summer losses out of 1399 colonies. As an effect of these low numbers, the modelled probabilities of summer loss stayed below 1% for all risk factors.

The loss of a queen raised the odds of summer loss by a factor of around 63 compared with a queenright colony (Table 3, P = 0.0059). The probability of the loss of a queenright colony in summer was approximately 0.01% (95%CI 0.00–0.10), when all other co-variables were set on mean. The probability for the loss of a queenless colony was 0.39% (95%CI 0.01–10.89). Odds of a weak colony to die in summer were 33 times higher than odds of a strong colony (Table 3, P = 0.003). Normally sized colonies and strong colonies showed no significant difference in probability of summer loss (P = 0.131). Odds for a summer loss increased by a factor of 1.17, when varroa mite infestation level increased by 1%. The model predicted a summer loss probability of 0.51% (95%CI 0.00-44.56%), when varroa mite infestation level was 30% (all other co-variates set on mean).

### Winter losses: Multivariate analysis

**Fig 4:**
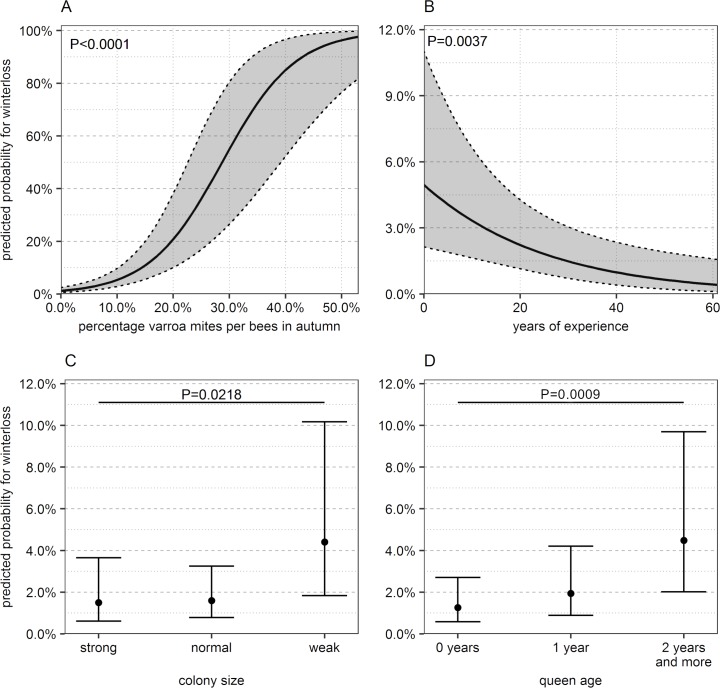
Marginal effects of probability for winter loss as predicted by the GLMM. Probability is shown for the four significant factors (A) varroa mite infestation level in autumn, (B) years of beekeeping experience, (C) estimated colony size in autumn and (D) age of the queen in autumn. For each graph the remaining co-variates are set to the mean. For detailed model see Table 4. Please note the different scales of the y-axis. n = 1382 colonies, N = 188 apiaries. Gray shadings, error bars: 95%CI.

The final GLMM model included the variables varroa mite infestation level in autumn, beekeeping experience, queen age and colony size in autumn (Table 4, Fig 4). The following variables were not included into the multivariate model, because the multivariate analysis showed no significant correlation with winter loss: type of colony in spring, varroa mite infestation level in summer, signs of Varroosis in summer and signs of Varroosis in autumn. The varroa mite infestation level in summer would have been a significant variable in the next step of the model. However, the Δ AIC was below 2 and thus indicated that the variable did not improve the overall model [47].

**Table 4:** Explanatory factors for winter losses in Austria in the winter season 2015/16.

Variable	Levels	Estimate (SE)	Odds (95% CI)	Z value	P
Intercept		-3.84 (0.57)	0.02 (0.01–0.07)	-6.69	**< 0.0001**
Mite infestation level autumn		0.15 (0.02)	1.17 (1.11–1.22)	6.19	**< 0.0001**
Years of experience		-0.04 (0.01)	0.96 (0.93–0.99)	-2.81	**0.0037**
Colony strength autumn reference level: strong	Normal	0.44 (0.32)	1.55 (0.83–2.90)	1.38	0.8653
	Weak	1.3 (0.39)	3.68 (1.70–7.97)	3.30	**0.0218**
Queen age autumn reference level: 0 year	1 year	0.06 (0.37)	1.06 (0.52–2.19)	0.16	0.1695
	>1 year	1.10 (0.48)	3.02 (1.17–7.75)	2.28	**0**.**0009**

GLMM with binomial distribution, random factor ‘apiary’. The random factor improved the model significantly (Chi^2^ = 71.82, df = 1, P < 0.001). n = 1382 colonies; N = 188 apiaries, SE = standard error.

Generally, the factors connected with winter loss can be divided in two classes of impact: The effect of varroa mite infestation level covered the whole range of winter loss probability from nearly 0% to up to 100% loss probability (Fig 4A). The other three factors had a smaller range of impact, which covered loss probability between 0% and 10% (Fig 4B–4D). Therefore the factor varroa mite infestation level had the power of raising the possibility of winter loss far higher than the other three variables.

A high varroa mite infestation level on bees in autumn raised the possibility of winter loss significantly (P < 0.0001, Table 4). When varroa mite infestation level in autumn was 0% the probability of winter loss was 1.2% (95%CI: 0.6–2.5%, Fig 4A), given that all other co-variates were set on mean. The possibility of winter loss remained below 10% up to a varroa mite infestation level of 10% (Fig 4). It increased steeply afterwards, raising the probability of loss to 54.6% (95%CI: 26.3–80.3%) at a varroa mite infestation level of 30%. When varroa mite infestation level reached 40% the probability of winter loss was more than 80%.

The possibility of winter loss decreased with increasing years of beekeeping experience (P = 0.0037, Table 4). However, the effect of one year of experience was low: the odds of winter loss decreased by a factor of 0.96 for each additional year of experience (Table 4). Colonies of beekeepers with one year of experience had a possibility of 4.7% winter loss (95%CI: 2.1–10.5%, Fig 4B), given that all other co-variates were set on mean. A beekeeper needed another 18 years of experience to reduce the possibility of winter loss by half (2.3% probability, 95%CI: 1.2–4.5%).

A young queen had a positive effect on the overwintering success. The odds that a colony died during winter increased by a factor of 3.02, when the queen was older than one year compared to a colony with a young queen (P = 0.0009, Table 4). There was no difference in the possibility of winter loss between colonies with new queens and colonies with queens of one year (P = 0.1695, Table 4).

A weak colony in autumn had a negative effect on the overwintering success in the following winter (Table 4). The odds to die increased by a factor of 3.68 in comparison to a colony, which was rated strong in autumn (P = 0.0218). There was no difference in winter loss probability between strong and normally sized colonies (P = 0.8653).

## Discussion

Our results give a first systematic and representative description of factors correlated with honey bee colony winter and summer losses in Austria. The data collection was focused on the beekeepers’ characteristics and the bee colonies’ health status. It demonstrates for the first time, that a high varroa mite infestation level of adult bees decreases survival rate of bee colonies not only in winter but already in summer. Generally, reasons for colony losses are similar in both seasons. However, the winter is the critical season for Austrian beekeepers, in which the health of colonies and beekeeping experience are the main factors for successful overwintering. Furthermore, our study provides data on the clinical prevalence of nine pests and diseases in summer, autumn and winter for Austrian bee colonies and apiaries.

### Colony losses

Our dataset shows that winter is the critical season for colony losses in Austria. In our study 75% of all colony losses were registered in winter. This loss pattern is also found in other European countries. A Norwegian study reports a similar proportion between summer losses and winter losses (7.7% losses in winter 2007/20018, 2.8% losses in summer 2008, [51]). The EPILOBEE study surveyed summer and winter losses from 17 countries from all over Europe and showed that the highest reported loss rates were reached during the winter season [17]. However, southern European countries such as France, Spain and Greece as well as surveys from the US reported similar loss rates in winter and summer [3, 4, 17, 52]. These results suggest that the relative importance of winter and summer mortality varies between different countries and climatic regions. Among others, the reasons for these differences may be found in differences between countries and climatic regions in land use [53, 54], in the amount of pesticide use [55–59], in the colonies’ population dynamics [60, 61] and in beekeeping practices[1, 2, 10, 31].

The winter colony loss rate derived from the field inspections of 1554 colonies in the investigated winter 2015/16 was 8.4%. This result can be compared to the longer ongoing COLOSS-studies on colony losses that collect a larger dataset on a voluntary base and at the level of beekeeping operations [8]. The winter loss rate for the same winter determined by such a survey covering 5% of all Austrian beekeepers was estimated to be 8.1% of 23 418 wintered colonies (95%CI 7.4–8.8%, [9]). However, the COLOSS-study asked for the beekeeping operations’ total winter losses, i.e. the winter losses of all apiaries. Additionally, the definition of winter loss differs between our study and the COLOSS-study, as the COLOSS-questionnaire added colonies, which are alive in spring, but lost because of unsolvable queen problems to the winter losses. When adding the queenless colonies in spring to the winter losses and calculating the losses based on the operation level losses, the losses of the surveillance study amount for 8.6% (95%CI 7.5–9.9%, for details see S8 Table), which is not different from the COLOSS results.

This very rare possibility to compare loss rates determined by a survey and a randomized field study suggests that sampling location selection in our surveillance study thoroughly represents Austrian beekeeping sector, or at least, that both studies addressed a similar subpopulation of beekeepers. Both findings also underline that the loss rate in the winter of the surveillance study ranked among the lowest recorded so far in Austria. We assume that this fact also strongly affected the disease prevalence and risk analysis of our study. During the winters before and after our surveillance study, a much higher loss was recorded in Austria by the COLOSS-survey (2014/15: 28% [10]; 2016/17: 23% [12]). A high variation in colony losses between successive years is a phenomenon observed not only in Austria but in countries throughout whole Europe [1, 9, 10, 12, 55]. This variation is not explained yet, but may arise from the variation in the prevalence of pests and diseases [6, 52, 55, 62] and their interaction with other risk factors such as climatic conditions, beekeeping practice or landuse [17, 63–65].

Before this study data about the amount of summer losses in Austrian bee colonies were lacking. Likewise, there exist surprisingly few data about summer losses in other European countries compared to the huge literature about winter losses. Furthermore, it is difficult to compare the few existing data, as the definition of summer loss or seasonal loss varies broadly between datasets. Sometimes the period from spring to summer is counted [5], sometimes the whole summer period [51, 66]. In this paper, we report yet another period of summer losses: late summer losses between July and September. The main reason for this lack of information may be that summer losses are difficult to track, because of colony splitting, swarming, merging or disposal of underperforming colonies, sales and transports, which are conducted or occur mostly in summer. However, the complexity of colony management in summer may also conceal existing problems in that season.

### Varroa mite infestation level

In our study the varroa mite infestation level was by far the most important factor for winter loss in Austria of those investigated. A varroa mite infestation level on adult bees over 40% in autumn increased the loss probability in winter to more than 80% (Fig 4A). In comparison, the other factors included in the model increased the probability of winter loss up to a maximum of 6% (Fig 4B–4D, years of beekeeping experience, colony size, queen age). Therefore, these factors had a much lower potential to raise the probability of colony loss.

A correlation of varroa mite infestation level with winter losses was shown before in several other European countries [5, 55, 67–69] and in North America [64, 70]. To our knowledge, only two other studies from the Netherlands measured varroa mite infestation level of adult bees in autumn and used this value for building a GLM with binomial distribution [69, 71]. The modelled slope of the first study describing the correlation between varroa mite infestation level in autumn and winter loss is similar to our model although those data are from another year (2011/2012) and another country (estimation Netherlands: 0.16 ± 0.06 standard error; our estimation: 0.15 ± 0.02 standard error; [69]). However, the second study from the Netherlands conducted in the years between July 2013 and April 2015 in one large apiary showed a steeper curve describing the relation between varroa mite infestation level and winter losses [71]. Unfortunately it did not give the estimate and standard error of the model. In that two-year study, a varroa mite infestation level of 5% led to a winter loss probability of 69%, which is a 28-fold loss probability compared to our model results for colonies with a similar infestation level. However, that study was conducted in one single apiary and therefore additional effects such as an unfavourable apiary location may have affected the colonies negatively independent of the varroa mite infestation level.

Treatment thresholds, which should not be exceeded to ensure a survival of the colony, are reported to be between 3 and 13% varroa mite infestation level on bees in autumn [72–74] and are low compared to the values of the correlation curve presented here. However, the aim of the presented study was to identify risk factors for colony losses and not to calculate a treatment threshold, which makes the study design not comparable to the mentioned studies with a clear experimental set-up (control groups without varroa treatment, even varroa mite distribution between the colonies, sister queens…). Furthermore, it would make sense to be very conservative in the calculation of a threshold, as the goal is to loose no colony at all because of Varroosis. Thus, one should set the threshold at a varroa mite infestation level, at which the probability of winter loss begins to rise (= the curve begins to rise). This condition is met in our curve at approximately 10% varroa mite infestation level–an infestation level similar to that in other studies [72–74].

We showed for the first time that varroa mite infestation level in July is correlated with late summer losses (losses between July and September). Chauzat et al. [5] analyzed factors correlated with seasonal mortality (colony losses between the spring visit and the summer visit) all over Europe and found no significant effect of varroa mite infestation level in spring. This is no contradiction to our results as they took samples in spring–i.e. some two to three months earlier in season than we did. Colonies, which are healthy enough to survive the winter, are expected to have a low varroa mite infestation level at the end of winter as survival is strongly correlated with a low varroa mite infestation level [75, 76]. This is also evident in our data, where clinical prevalence of Varroosis is significantly less frequent in spring than in summer and autumn of the previous year (Fig 2A). After the low winter level, the varroa mite population needs time to rise to a critical level [77] and most colonies reach a critical infestation level as late as August [62]. However, our results demonstrate that a critical threshold can be reached by the end of July and consequently leads to late summer losses due to that critical level of varroa mite infestation.

Modelling revealed that the best *Varroa* related predictor for colony losses is the varroa mite infestation level of bees shortly before the loss period. Other tested variables connected with the varroa mite predicted colony losses significantly in univariate models, but not in the multivariate model (Tables 2–4). For example, Varroosis was significant in univariate testing of both summer and winter loss, but it was not included into the two multivariate models. Instead varroa mite infestation level was included, because it described the harmful effect of the varroa mite more accurately. Similarly, varroa mite infestation level in summer was significantly correlated with winter loss, but did not explain the loss probability as well as varroa mite infestation level in autumn.

### Queen age and queen failure

We showed that the queen is a significant factor in estimating the loss probability in both summer and winter (Tables 3 and 4). However, the nature of the queen problem differed between the seasons: the significant factor in summer was ‘queen failure’, while in winter ‘queen age’ was significantly affecting colony loss.

We demonstrated that a colony had a higher risk of summer loss, when it was queenless in July. This indicates, that time of queen replacement is a sensitive phase in colony development. During this phase, problems with requeening may occur (e. g. loss of the queen during mating flight or during hive manipulations, unsuccessful mating, introduction of a weak/ill queen, rejection of the new queen by the worker bees). Each of these events leads to a malfunctioning colony with no or a reduced production of worker bees and finally to the death of the colony.

In winter, colony loss was significantly increased, when the queen was older than one year. A correlation between queen age and the probability of winter loss was also found in other European and American countries [2, 55, 78]. Old age in queens seems to be connected with certain physiological deficits which have a negative impact onto colony development and health and which may lead to weakened colonies with an increased probability of winter loss. For example, old queens are correlated with reduced worker production and small colony size [79–81]. Generally, worker bees of colonies with old queens have a lower probability to survive the winter [79]. Furthermore, studies suggest that colonies headed by an old queen are infested more severely with varroa mites than colonies headed by a young queen [82, 83].

### Colony size

Our data show, that small colony size is not only a predictor for winter loss, but also for summer loss. The correlation between winter loss and colony size is a frequent pattern, which was reported also from other European and North American countries [55, 67, 70]. Generally, small colony size is an unspecific characteristic which correlated in our dataset with a range of different factors (S5 Table). Two factors correlated with small colony size were identified by the model as influential factor and are thus already included in the model (varroa mite infestation = Varroosis, queen failure). Also, certain management practices such as colony splitting, nuc installation, installation of a new queen and brood removal are correlated with small colony size. Our data set shows no evidence that these practices are connected with an increased probability of winter loss (Table 2, S5 Table).

A range of unspecific brood symptoms such as patchy brood pattern, punctured cell cappings and dead brood were also correlated with small colony size (S6 Table). These symptoms hint towards problems with brood rearing such as a reduced queen’s egg laying rate [80] or brood loss through pesticide exposure or brood diseases and pests [18, 21, 25, 27, 84, 85]. The factors diseases and pesticides may have led also to a decline of adult worker bees and thus to a depopulation of the colony [71, 75, 86–89], which in turn results in problems with brood rearing through the lack of sufficient nurse bees [90].

We showed that a simple classification of colony size into three categories (very small, normal sized, very big) allowed predicting the risk of loss. In previous works, colony size was determined exactly and time-consuming by estimating the number of bees per colony [55, 67, 70]. In contrast, we instructed the bee inspectors to assess, whether the bee colony was normal sized as would be expected for the particular region and time of the year or whether it was exceptionally small or exceptionally big. It seems that an approximation from an experienced bee inspector has a similar prediction value of winter loss as an exact measurement of the bee population.

### Beekeeping experience

Long-time experience in beekeeping significantly reduced the probability of winter colony losses. EPILOBEE found a significant positive relationship between the beekeepers’ age and winter loss [5]. However, in their dataset highest winter losses occurred in apiaries of old beekeepers with few colonies and low beekeeping experience [17]. In contrast, in our study the beekeepers with more years of experience (= older beekeepers) had more bee colonies than beginners in beekeeping. A high number of colonies can be regarded as sign for a high level of professionalism [1, 3]. Therefore results of both studies show consistently, that European beekeepers with a high level of professionalism (high number of colonies, long experience as beekeeper) lost fewer colonies than beekeepers with a low level of professionalism. The same results were reported by two studies from the USA [3, 4].

We included further factors to evaluate professionalism such as apiary size, operation size or highest beekeeping education. However, all these factors showed no correlation with losses or were not included into the multivariate model, because they did not improve the explanatory power of the model. The reason for the insignificance of these variables in our study may be the relatively small sample size of 189 beekeepers. Studies, which showed a relation between colony loss and operation size, involved several thousand datasets [2, 3, 5]. Other survey-based studies with larger datasets confirmed this trend also for Austria [8, 10].

### American foulbrood

In our dataset clinical symptoms of AFB were significantly related to an increase in summer loss (Table 2, S2 Table). However, it was reported for only one apiary in summer and was therefore too rare to be significant in the multivariate model. Nevertheless, we want to clarify that AFB was a lethal disease in our study [18]. Our results are consistent with the results of the EPILOBEE-study, in which clinical signs of AFB were described as main risk factor for winter loss together with clinical signs of Varroosis and Nosemosis [5].

### Random effect of the GLMMs

It is necessary to emphasize the fact, that both GLMM-models included the apiary identity as random effect, which was in both models significant (Tables 3 and 4). This signifies that part of the variation in colony losses between the apiaries is not explained–i.e. factors, which are needed to explain that variation, are missing in the models. Possible influential characteristics of the apiary onto probability of colony loss, which are not evaluated in our study, are available natural food sources and their quality [64, 65, 91], pesticide exposure within the flight range [2, 69], climatic conditions of the apiary [60, 63], level of local adaptation of the queens’ genotype [92], mite reinvasion from neighboring colonies or apiaries after sampling [93, 94], quality and quantity of winter food [95–97] and prevalence and infestation level of bee viruses in the apiary [55, 67, 98]. Next to the recorded and already discussed beekeepers characteristics, the variation on apiary level can also be affected by not recorded or not recordable beekeeper properties, such as hive management, pest control and their suitability for the particular climate, location or weather conditions [1, 2, 99].

### Prevalence of diseases

During one year of surveillance, clinical signs of seven brood and bee diseases were reported for Austria’s apiaries. The brood diseases AFB, Chalkbrood and Sacbrood were observed most frequently during spring and summer, when colonies have the largest brood areas in their nests. The parasitic disease Varroosis, which affects both brood and adult bees, was reported most frequent in summer and autumn. CBPV and Nosemosis are diseases of the adult bee. Symptoms of these two diseases were generally seldom and showed no seasonal pattern. EFB and the two bee parasites *A*. *tumida* and *Tropilaelaps* spp. were not observed during the surveillance study.

To make data internationally comparable, data collection about prevalence in bee diseases complied in large parts with the instructions for the EPILOBEE project [17, 100]. The main differences were that we performed no laboratory analysis in case of Varroosis and that we surveyed additionally for the two bee diseases Chalkbrood and Sacbrood. The validity of the on-site Varroosis diagnosis is confirmed by the significant correlation between the Varroosis diagnosis and the varroa mite infestation rate (Fig 3B).

Among the observed diseases, clinical Varroosis was most frequently reported (spring: 8% of the apiaries, summer and autumn: 22% of the apiaries). This result is in congruence with the results from the first year of EPILOBEE, showing that in all 17 participating European countries Varroosis was the most commonly reported disease (prevalence in autumn 14.9% of the apiaries, in spring 14.8% of the apiaries; [5]).

In Austria, Varroosis was significantly more frequent in summer and autumn than in spring. This corresponds with the population dynamics of the varroa mite in a honeybee colony, which is increasing from spring to autumn and decreasing in winter [101]. Besides, bees with Varroosis symptoms seem to have an increased probability to die during winter due to the coinfection with Deformed Wing Virus and therefore the frequency of Varroosis symptoms decrease in the bee population over winter [102].

AFB was found rarely during the study. It was detected in one apiary in spring and one apiary in summer; in autumn no symptoms were found in any apiary (prevalence level per visit: 0.0–0.6%). Therefore, clinical prevalence of AFB was lower in Austria than in the dataset from EPILOBEE (prevalence autumn: 2.4%, spring 1.9%; [5]). AFB is a notifiable disease in Austria [31]. In the years between 2007 and 2016 a mean of 143 ± 32 s.d. newly infected apiaries per year were registered in Austria [103]. Unfortunately, no information is available about the total number of apiaries in Austria to calculate a frequency of outbreaks. However, we know that 26 609 beekeepers were registered in the two national beekeeping associations in 2016 [104], which means that at least 26 609 apiaries were located in Austria in 2016. Conclusively, a mean of 0.5% of Austrian beekeepers are confronted with AFB-outbreaks per year–this rough estimate matches our results quite well.

Our results confirm that Austria is still free from *Tropilaelaps spp*. and *A*. *tumida*. However, global trade and climate change facilitate the distribution of these bee parasites and it is possible that they may be introduced to Austria in future [105, 106]. *A*. *tumida* was already introduced twice into Europe: in 2004 two living larvae of *A*. *tumida* were discovered in queen cages carrying honey bee queens during an official import into Portugal, but a successful introduction was prevented by destruction of the destination apiary [107]. In the year 2014, it was introduced into Europe a second time at the port Gioia Tauro in Southern Italy; its occurrence is still limited to the South of Italy [108]. Currently, the distribution of *Tropilaelaps spp*. is limited to Asia [109]. *Tropilaelaps* mites depend on honey bee brood to feed on the larvae and survive only a few days without brood [110]. Nevertheless, they may be able to survive a transportation via plane to Europe, which makes an introduction via package bees, shipped queens or bee hive products possible [105, 111].

EFB was not detected in the recent study. Our result is in congruence with the experience of the Austrian reference laboratory for honeybee health located in the AGES, where only three cases of EFB were confirmed between 2006 and 2013 (Derakhshifar, unpublished data). However, EFB is no notifiable disease in Austria [31] and therefore the reference laboratory may not have knowledge of all cases of EFB in Austria. Generally, EFB is seldom throughout Europe as the prevalence during the first year of EPILOBEE accounted for 1% of the apiaries [5]. It appears regularly only in some European countries such as France, Switzerland, Spain and Italy [52, 112–114].

Clinical signs of the two brood diseases Chalkbrood and Sacbrood were reported second and third most frequent in Austrian apiaries after Varroosis. These diseases were not included into the observation scheme of the EPILOBEE study; therefore we cannot compare our results to other European clinical prevalence data. However, prevalence of the Sacbrood Virus (SBV) is frequent in European honeybee colonies. It was present in samples from about 33% of the colonies in an Italian surveillance study [6], in up to 15% of samples in a German surveillance study [55] and in up to 64% of the samples in a French surveillance study [115]. The presence of SBV in Austria ranged between 14% and 50% in dead or diseased colonies [14, 116] and up to 75% in inconspicuous colonies [15]. Historic surveillance data of SBV in Austria showed generally higher prevalence values than our results of clinical prevalence of Sacbrood (between 0.2 and 1.3% of all colonies, S7 Table). However, the prevalence of the virus is generally higher than the prevalence of the clinical symptoms, because the Sacbrood Virus can be present in a colony as covert as well as overt infection [117].

Clinical cases of CBPV and Nosemosis were rare and reached both a maximum of 1.1% prevalence in Austrian apiaries. CBPV was also rarely found in the EPILOBEE project (prevalence apiaries in autumn: 0.7%, in spring: 0.9%; [5]). Although it is seldom, CBPV-infection is connected with health problems in Austrian bee colonies. In the years 2003/2004 bee samples from 90 colonies with diverse symptoms were tested for bee viruses; 10% of these samples were positive for CBPV and showed typical symptoms for CBPV [14]. In spring, Nosemosis was found more frequently in the EPILOBEE-study than in our Austrian results (prevalence autumn: 0.6%, spring: 8.9%; [5]). However, in both studies covert infections with *N*. *ceranae*, which are visible only by the symptom of depopulation, may have been missed and therefore the prevalence may be underestimated [38].

## Conclusions

Generally, the inspected Austrian honeybee colonies were in good health during the time period of the study. Clinical symptoms of only three diseases were found in more than 5% of the apiaries. Two of these diseases–Chalkbrood and Sacbrood–seemed to be no immediate threat to the colonies as they were not connected with colony losses. In contrast Varroosis was the only disease, which was both frequently present in the colonies and correlated significantly with summer and winter colony losses, respectively. However, further studies should additionally focus on the detection of diseases with inconspicuous symptoms to ensure the detection of diseases responsible for the weakness of some colonies.

Reasons for colony losses were mostly similar in late summer and in winter. Thus, beekeepers should pursue the same strategy in summer and in winter: keep strong colonies with a young and healthy queen and control the level of varroa mites by accurately timed and effective control measures. Additionally, a long time experience in beekeeping is helpful to keep winter losses low. When ranking the significant factors, it shows that the varroa mite is by far the most influential factor. Thus, control of the mite should have the highest priority in order to reduce colony losses. The significant factor with the least effect on losses was the beekeeping experience, expressed as years of beekeeping activity. However, it has to be considered that the study was conducted in a season with extremely low winter losses. In the preceding and the following winter seasons the colony losses were three times as high as in the study winter. Thus, bee health may have been worse and clinical prevalence may have been higher in these years. Similarly, reasons for winter losses and the strength of their impact may differ in years of high losses (e.g. a high level of beekeeping experience may be more advantageous). Multiyear surveys and surveillance studies are necessary to solve these questions and to detect new emerging problems.

## Supporting information

S1 Text Selection of the participating beekeepers. (PDF)

S2 Text Survey questionnaire in German (first visit). (PDF)

S3 Text Survey questionnaire translated to English (first visit). (PDF)

S4 Text Effect of missing cases on modelling of colony losses. (PDF)

S1 Dataset GPS-coordinates of the 189 apiary locations. The GPS-coordinates were rounded to 2 decimal places to ensure privacy of the beekeepers (mean deviation from location: 345m ± 142 s.d.).
(CSV)

S1 Table Primers and PCR-parameters for the tests for *Nosema apis*, *Nosema ceranae* and CBPV. (PDF)

S2 Table Clinical prevalence of six bee diseases and colony losses. Correlation between clinical prevalence of six diseases in the observed 1596 colonies and survival of the colonies in summer 2015 (between July 2015 –September 2015) and the winter season 2015/16, respectively. The percentage of dead colonies per group (= row) is given in brackets. Each variable is tested with a Chi^2^-Test or a Fisher’s Exact Test (FET), respectively. Diseases without positive cases were not tested and are therefore not shown. Significant results are shaded in gray.
(PDF)

S3 Table Categorical colony characteristics related with survival in summer 2015 (summer losses) and winter 2015/16 (winter losses), respectively. The percentage of dead colonies per group (= row) is given in brackets. Each variable is tested with a Chi^2^-Test or a Fisher’s Exact Test (FET), respectively. Number of missing values for each Variable and testing periods are given. n = 1569 colonies.
(PDF)

S4 Table Metric colony characteristics related with survival in summer 2015 (summer losses) and winter 2015/16 (winter losses), respectively. For each variable the number of cases (N), the median and the first (Q1) and third quartile (Q3) are given. Differences between the groups of alive and dead colonies were tested with a Wilcoxon Rank Sum Test (significant differences highlighted in gray). cols = colonies, yrs = years. n = 1569 colonies.
(PDF)

S5 Table Correlation between small size of the colonies and colony characteristics, diseases. The percentage of weak colonies per group (= row) is given in brackets. Each variable is tested with a Chi^2^-Test or a Fisher’s Exact Test (FET), respectively. Significant results are shaded in gray.
(PDF)

S6 Table Correlation between small size of the colonies and disease symptoms. The percentage of weak colonies per group (= row) is given in brackets. Each variable is tested with a Chi^2^-Test or a Fisher’s Exact Test (FET), respectively. Significant results are shaded in gray.
(PDF)

S7 Table Prevalence of bee diseases and pests in the study apiaries and colonies. 95%CI is given in brackets. Prevalence was evaluated by checking the bee colonies for clinical signs (Table 1). Cases with signs of American Foulbrood, CBPV and Nosemosis were confirmed by using standard laboratory tests. Sampling was done in summer 2015 (July, August), autumn 2015 (September, October) and spring 2016 (March-May).
(PDF)

S8 Table Winter losses of study colonies, participating apiaries (N = 188) and participating operations (N = 188). The losses are calculated for two different definitions of winter loss: (1) dead colonies and (2) dead colonies and living colonies with unsolvable queen problems in spring. 95%CI = 95% Confidence Interval.
(PDF)
