# Supplementary material for: Health status of honey bee colonies (Apis mellifera) and disease-related risk factors for colony losses in Austria
Source: PLoS One. 2019 Jul 9;14(7):e0219293. doi: 10.1371/journal.pone.0219293 (PMC6615611; doi:10.1371/journal.pone.0219293)
Supplement: S1 Text — (PDF) [file pone.0219293.s001.pdf]

S1 Text. Selection of the participating beekeepers

The ideal sampling scheme for the study would have been a random sampling from the whole population of apiaries (van der Zee et al. 2013). Unfortunately, at the start of the project Austria had no national register of beekeepers or apiaries. Therefore, a random sampling of participants was not possible. Additionally, this lack of an register meant also a knowledge gap of the total number of beekeepers and apiaries in Austria.

Thus, we relayed in our planning on the data of the two beekeeping associations (Austrian Beekeepers Association, Austrian Professional Beekeepers Association), in which most Austrian beekeepers are registered. However, these data were not available to us for reasons of data protection. To overcome this problem, we produced a randomly selected list of postal codes, which reflected the distribution of members of the Austrian Beekeepers Association over Austria (Fig 1). Then we asked the Austrian Beekeepers Association to search for beekeepers with a home address matching the selected postal codes and ask them to participate in the study. This system worked well for six federal states (54 % of the 143 beekeepers). In two federal states no structured data bases were run by the local branch of the Austrian Beekeepers Association. Therefore, they advertised the project among their members and participants were chosen randomly from the list of volunteers (32 % of the 143 beekeepers). In one federal state, the Beekeepers Association provided a list of participants, which matched only partly with the randomly chosen list of postal codes (14 % of the 143 beekeepers).

In total 12 beekeepers dropped out of the project; one of them could be replaced before the start of the project. The reasons for dropping out were problems with time management (4 beekeepers), health issues of the beekeeper (3 beekeepers), loss of all bee colonies during the project due to a fire (1 beekeeper), apiary location in a zone under quarantine due to an outbreak of American Foulbrood (1 beekeeper) and disapproval with the methodology of the surveillance study (1 beekeeper). Two beekeepers dropped out of the study without any further explanatory statement.

References

van der Zee R, Gray A, Holzmann C, Pisa L, Brodschneider R, Chlebo R, et al. Standard survey methods for estimating colony losses and explanatory risk factors in *Apis mellifera*. In: Dietemann V, Ellis JD, Neumann P, editors. The COLOSS BEEBOOK, Volume I: Standard methods for *Apis mellifera* research. J Apic Res. 2013;52(4).
