# Supplementary material for: Health status of honey bee colonies (Apis mellifera) and disease-related risk factors for colony losses in Austria
Source: PLoS One. 2019 Jul 9;14(7):e0219293. doi: 10.1371/journal.pone.0219293 (PMC6615611; doi:10.1371/journal.pone.0219293)
Supplement: S2 Text — (PDF) [file pone.0219293.s002.pdf]

1. Bienenstandbesuch, Juli 2015

[Bienenstand Nr: W_03]

PROBENNEHMER: Köglberger **Datum*:** ....

Mit Sternchen* gekennzeichnete Felder müssen unbedingt ausgefüllt werden!

Generelle Information zum Imkereibetrieb und zum Bienenstand:

1/ ImkerIn

Hans Imkermeister
Honigstraße 52a
1234 Bienenfeld

LISA Etikett groß

Tel: ....

E-Mail: ....

Gesamtzahl aller Bienenstände des Betriebs zum heutigen Datum*: ....

Gesamtzahl aller Bienenvölker des Betriebs zum heutigen Datum *: ....

Ist der Betrieb ein zertifizierter Biobetrieb?* ☐ ja ☐ nein

Betriebsziel des Imkereibetriebes? (Mehrfachnennung möglich)

☐ Honigproduktion ☐ Jungvolkproduktion ☐ Königinnenproduktion

ImkerIn seit dem Jahr: ....

Ausbildungen: ☐ keine ☐ Grundkurs ☐ Aufbaukurse ☐ FacharbeiterIn ☐ ImkermeisterIn ☐ WanderlehrerIn

Interesse an Fortbildungen: ☐ ja ☐ nein

Wie häufig verwenden Sie folgende Informationsquellen zu Fortbildungszwecken?

	mind. einmal pro Monat	mehrmals jährlich	einmal im Jahr	alle paar Jahre	nie
Kurse, Schulungen	☐	☐	☐	☐	☐
Vorträge, Imkereitagungen	☐	☐	☐	☐	☐
Vereinsabende	☐	☐	☐	☐	☐
Imkerzeitschriften	☐	☐	☐	☐	☐
Internet, Imkerforen	☐	☐	☐	☐	☐

2/ Besuchter Bienenstand

Standort: PLZ*: .... Standgemeinde*: ....

Katastralgemeinde*: ....

GPS (6 dezimale):

Breitengrad*:....................................... - Längengrad*: .... (z.B.: 43.612454 -7.054878)

Seehöhe*:..........................

Welche Art der Landnutzung gibt es im 3km-Umkreis des Bienenstandes (ungefähre Schätzung)?

- ☐ Ackerflächen: ....Prozent
- ☐ Wiesenflächen: ....Prozent
- ☐ Waldflächen: ....Prozent
- ☐ Siedlungsräume: ....Prozent

Sind Sie mit den Völkern dieses Bienenstandes in dieser Saison gewandert? ☐ Ja ☐ Nein

Wenn Ja; Wanderungen 2015:

Tracht	Land/Bundesland	Datum Anwanderung	Datum Abwanderung

3/ Risiko-Faktor für die Einschleppung exotischer Tiere

Wurden seit Anfang 2013 Königinnen für diese Imkerei nach Österreich importiert?

☐ Ja ☐ Nein

Falls ja, Angabe des oder der Herkunftsländer: ....

Wurden seit Anfang 2013 Bienenvölker, Schwärme oder Paketbienen für diese Imkerei nach Österreich importiert?

☐ Ja ☐ Nein

Falls ja, Angabe des oder der Herkunftsländer: ....

4/ Völkerbestandsentwicklung in der Saison 2014/2015

a) Überwinterung an diesem Bienenstand

- Anzahl eingewinterter Völker 2014*: ....
- Anzahl ausgewinterter Völker 2015*: ....

b) Zu diesem Bienenstand 2015 bisher hinzugekommene Völker*: ....(insg.)

- Davon zugekauft oder von anderem eigenen Stand verbracht*: ....
  (falls nicht aus Österreich, Herkunftsland: ....)
- Davon Ableger*: ....
- Davon natürliche Schwärme und Kehrschwärme*:........................

c) Aus diesem Bienenstand seit der Auswinterung 2015 weggefallene Völker*: ....(insg.)

- Davon verkauft oder an einen anderen Stand gebracht*: ....
- Davon weggefallen durch Vereinigung mit einem anderen Stock*: ....
- Davon gestorben*:........................

d) In diesem Bienenstand 2015 neu eingesetzte Königinnen*: .... (insg.)

- Davon gekauft*:.................. (falls nicht aus Österreich, Herkunftsland: ....)
- Davon selbst produziert*: ....

5/ Angaben zu allen vorgenommenen medikamentösen Behandlungen an diesem Stand*

Bitte führen Sie – wenn möglich – alle in den letzten 12 Monaten vorgenommenen Behandlungen auf. Angaben so genau wie möglich!

Produktname od. Wirkstoff (inklusive Konzentration)	Dosierung pro Volk	Applikationsart	Datum der Behandlungen	Dauer der Behandlung

6/ Angaben zu allen vorgenommenen biotechnische und anderen Methoden an diesem Stand*

Bitte führen Sie – wenn möglich – alle in den letzten 12 Monaten vorgenommenen Behandlungen auf. Angaben so genau wie möglich!

Art der Behandlung	durchgeführt	Datum der Behandlung				
**Drohnenbrutentnahme**	☐ Ja ☐ Nein					
**Wärmebehandlung – gesamter Stock**	☐ Ja ☐ Nein<br>Gerät......................					
**Wärmebehandlung – Brutwaben**	☐ Ja ☐ Nein<br>Gerät......................					
**Gesamtbrutentnahme**	☐ Ja ☐ Nein					
**Brutunterbrechung**	☐ Ja ☐ Nein					
**Bannwabenverfahren**	☐ Ja ☐ Nein					

7/ Winterverluste 2014 / 2015

Dieser Abschnitt behandelt die Winterverluste ***ALLER Völker des Imkerbetriebs*** *und ist nicht nur auf den beprobten Stand bezogen.*
Die Fragen stammen von der Internetplattform „bienenstand.at" der Universität Graz, die auch ein Teil des Projekts „Zukunft Biene" darstellt. Sie sollen auch beantwortet werden, wenn der Imker / die Imkerin schon an der Umfrage der Universität Graz teilgenommen hat.

Hat sich der Imker / die Imkerin an der Erhebung der Winterverluste 2014/2015 der Uni Graz beteiligt (Bienenstand.at)?* ☐ Ja ☐ Nein

In welchem Bezirk stehen die Bienenvölker?*

☐ im Bezirk:..

☐ in mehreren Bezirken

Wo befindet sich der (Haupt-)Bienenstand (Winterstandort)? Bitte Gemeinde und PLZ des Standortes nennen.*

Gemeinde..; PLZ: ....

An wie vielen verschiedenen Standorten (Anzahl) werden Bienen gehalten?*......................

Werden <u>alle</u> Bienen innerhalb von etwa 15 km des genannten Standortes gehalten?*

☐ Ja ☐ Unsicher ☐ Nein

Wie viele Bienenvölker wurden 2014 auf allen Ständen eingewintert?*..............

Wie viele dieser Völker gingen im Winter 2014/2015 auf allen Ständen verloren?*

- Tote oder verlorene (leere) Völker? ....
- Durch Königinnenprobleme ....

Basisdaten zu und Beobachtungen an den Fokus-Völkern

BITTE ENTSPRECHEND AUSFÜLLEN

**A) Beschreibung des Volkes** *	**Nummern der Fokus-Völker**											
	**1**	**2**	**3**	**4**	**5**	**6**	**7**	**8**	**9**	**10**	**11**	**12**
Königin stammt aus dem Jahr…												
Wirtschaftsvolk (**WV**) oder Jungvolk (**JV**)												
Jungvolk: Kehrschwarm (**KS**) oder Ableger (**A**)												
Volk in diesem Jahr gewandert: ja (J) oder nein (N)												
Wabenmaß (wenn unterschiedlich: Brutraum + Honigraum)												
Anzahl Zargen												
Anzahl Waben pro Zarge												
Volksstärke (1: sehr schwach; 2: normal; 3: sehr stark)												

BITTE ZUTREFFENDES ANKREUZEN:

**B.1) Beobachtete Symptome vor Öffnen***	**1**	**2**	**3**	**4**	**5**	**6**	**7**	**8**	**9**	**10**	**11**	**12**
Tote Bienen vor dem Bienenvolk	☐	☐	☐	☐	☐	☐	☐	☐	☐	☐	☐	☐
Lebende, flugunfähige Bienen am Boden	☐	☐	☐	☐	☐	☐	☐	☐	☐	☐	☐	☐
Kot / Kotflecken	☐	☐	☐	☐	☐	☐	☐	☐	☐	☐	☐	☐
Zitternde Bienen	☐	☐	☐	☐	☐	☐	☐	☐	☐	☐	☐	☐
Schwarz-glänzende Bienen	☐	☐	☐	☐	☐	☐	☐	☐	☐	☐	☐	☐
Zurückgewiesene Bienen	☐	☐	☐	☐	☐	☐	☐	☐	☐	☐	☐	☐
Ausgetragene Brut	☐	☐	☐	☐	☐	☐	☐	☐	☐	☐	☐	☐
Verstopftes Flugloch	☐	☐	☐	☐	☐	☐	☐	☐	☐	☐	☐	☐
Anderes (Fußnote setzen u. unten beschreiben)												

Bitte beschreiben Sie außergewöhnliche Beobachtungen und Symptome (eventuell mit Fußnotenbezug):
z. B.: Verwendung von Hinterbehandlern, tote Völker am Stand,…

Abkürzungen Wabenmaß:
Z.....................Zander
ZF ....Zander flach
DN = EM ....Deutschnormal, Einheitsmaß
BW..................Breitwabe
D.....................Dadant
Wenn anderes Maß verwendet, Abkürzung hier vermerken:

Basisdaten zu und Beobachtungen an den Fokus-Völkern

BITTE ZUTREFFENDES ANKREUZEN:

**B.2) Beobachtete Symptome im Volk***	**1**	**2**	**3**	**4**	**5**	**6**	**7**	**8**	**9**	**10**	**11**	**12**
Lückiges Brutbild	☐	☐	☐	☐	☐	☐	☐	☐	☐	☐	☐	☐
Löchrige Zelldeckel	☐	☐	☐	☐	☐	☐	☐	☐	☐	☐	☐	☐
Eingesunkene Deckel / abweichende Färbung	☐	☐	☐	☐	☐	☐	☐	☐	☐	☐	☐	☐
Zusammengesunkene Larven	☐	☐	☐	☐	☐	☐	☐	☐	☐	☐	☐	☐
Verfärbte Larven / Puppen	☐	☐	☐	☐	☐	☐	☐	☐	☐	☐	☐	☐
Abgestorbene Brut	☐	☐	☐	☐	☐	☐	☐	☐	☐	☐	☐	☐
Typischer AFB Geruch	☐	☐	☐	☐	☐	☐	☐	☐	☐	☐	☐	☐
Fadenziehende Masse	☐	☐	☐	☐	☐	☐	☐	☐	☐	☐	☐	☐
Schorfe	☐	☐	☐	☐	☐	☐	☐	☐	☐	☐	☐	☐
Sackbrutsymptome (Säckchen, Schiffchen)	☐	☐	☐	☐	☐	☐	☐	☐	☐	☐	☐	☐
Kalkbrutmumien	☐	☐	☐	☐	☐	☐	☐	☐	☐	☐	☐	☐
Deformierte Flügel	☐	☐	☐	☐	☐	☐	☐	☐	☐	☐	☐	☐
Varroamilben auf Bienen	☐	☐	☐	☐	☐	☐	☐	☐	☐	☐	☐	☐
Varroamilben in Zelldeckeln	☐	☐	☐	☐	☐	☐	☐	☐	☐	☐	☐	☐
Verdächtige Milben (außer Varroa)	☐	☐	☐	☐	☐	☐	☐	☐	☐	☐	☐	☐
Verdächtige Eier / Larven / Käfer	☐	☐	☐	☐	☐	☐	☐	☐	☐	☐	☐	☐
Anderes (Fußnote setzen + unten beschreiben)												

**C) Verdacht auf***	**1**	**2**	**3**	**4**	**5**	**6**	**7**	**8**	**9**	**10**	**11**	**12**
Varroose	☐	☐	☐	☐	☐	☐	☐	☐	☐	☐	☐	☐
Amerikanische Faulbrut (Verdachtsproben nehmen!)	☐	☐	☐	☐	☐	☐	☐	☐	☐	☐	☐	☐
Europäische Faulbrut (Verdachtsproben nehmen!)	☐	☐	☐	☐	☐	☐	☐	☐	☐	☐	☐	☐
Sackbrut	☐	☐	☐	☐	☐	☐	☐	☐	☐	☐	☐	☐
Kalkbrut	☐	☐	☐	☐	☐	☐	☐	☐	☐	☐	☐	☐
Nosemose (Verdachtsproben nehmen!)	☐	☐	☐	☐	☐	☐	☐	☐	☐	☐	☐	☐
Chronische Bienenparalyse (Verdachtsproben nehmen!)	☐	☐	☐	☐	☐	☐	☐	☐	☐	☐	☐	☐
Flügeldeformationsvirus (DWV) (Verdachtsproben nehmen!)	☐	☐	☐	☐	☐	☐	☐	☐	☐	☐	☐	☐
Kleiner Beutenkäfer (Verdachtsproben nehmen!)	☐	☐	☐	☐	☐	☐	☐	☐	☐	☐	☐	☐
Tropilaelaps-Milben (Verdachtsproben nehmen!)	☐	☐	☐	☐	☐	☐	☐	☐	☐	☐	☐	☐
Anderes (Fußnote setzen u. unten beschreiben)												
**Totes Bienenvolk (nur bei Besuch 2 und 3)**	☐	☐	☐	☐	☐	☐	☐	☐	☐	☐	☐	☐

Bitte beschreiben Sie außergewöhnliche Symptome oder Krankheiten (mit Fußnotenbezug):

Liste der genommenen Proben

**PROBENNAHME***	Nummer der Fokus-Völker						
	**1**	**2**	**3**	**4**	**5**	**6**	**7**
**Pflichtproben – Probennummer (letzte 3 Stellen der Etikettennummer) eintragen**							
Bienenprobe Varroa<br>großer Becher<br>**Bei Besuch 1 und 2**							
Bienenprobe PostHoc<br>Kleiner Becher<br>**Bei allen Besuchen**							
Bienenbrot-Probe<br>**Bei allen Besuchen**							
**Verdachtsproben – passendes LISA-Etikett aufkleben oder Probennummer (letzte 3 Stellen der Etikettennummer) eintragen**							
Bienen –<br>außerhalb gesammelt							
Bienen –<br>innerhalb gesammelt							
Verdächtige Brutwabe							
Verdächtige Milben							
Verdächtige Käfer / Käferlarven							
Anderes (bitte beschreiben)							

Liste der genommenen Proben

PROBENNAHME*	Nummer der Fokus-Völker						
	8	9	10	11	12	13	14
**Pflichtproben – Probennummer (letzte 3 Stellen der Etikettennummer) eintragen**							
Bienenprobe Varroa<br>großer Becher<br>**Bei Besuch 1 und 2**				X	X	X	X
Bienenprobe PostHoc<br>Kleiner Becher<br>**Bei allen Besuchen**				X	X	X	X
Bienenbrot-Probe<br>**Bei allen Besuchen**				X	X	X	X
**Verdachtsproben – passendes LISA-Etikett aufkleben oder Probennummer (letzte 3 Stellen der Etikettennummer) eintragen**							
Bienen –<br>außerhalb gesammelt							
Bienen –<br>innerhalb gesammelt							
Verdächtige Brutwabe							
Verdächtige Milben							
Verdächtige Käfer / Käferlarven							
Anderes (bitte beschreiben)							
