# Supplementary material for: Health status of honey bee colonies (Apis mellifera) and disease-related risk factors for colony losses in Austria
Source: PLoS One. 2019 Jul 9;14(7):e0219293. doi: 10.1371/journal.pone.0219293 (PMC6615611; doi:10.1371/journal.pone.0219293)
Supplement: S3 Text — (PDF) [file pone.0219293.s003.pdf]

1st Visit, July 2015

[Apiary ID: W_03]

Surveillance inspector: [Surname] **Date*:** ....

* mandatory fields!

General information about the beekeeping operation (=bk operation) and the visited apiary:

1/ Beekeeper

Hans Imkermeister
Honigstraße 52a
1234 Bienenfeld

[Barcode-label with sample number]

Phone: ....

E-Mail: ....

Total number of all apiaries of the bk operation at the current date*: ....

Total number of all bee colonies of the bk operation at the current date*: ....

Is the operation certified as 'organic' bk-operation?* ☐ yes ☐ no

Business objective of the bk operation? (multiple answers possible)

☐ honey ☐ package bees ☐ queens

Beekeeper since the year: ....

Beekeeping education: ☐ none ☐ beginner's course ☐ advanced courses
☐ skilled worker ☐ beekeeping master ☐ beekeeping instructor

Interest in further training: ☐ yes ☐ no

How often do you use the following information sources for advanced education?

	At least once a month	Several times a year	Once a year	Every few years	never
Courses, educations	☐	☐	☐	☐	☐
Talks, conferences	☐	☐	☐	☐	☐
Club evenings	☐	☐	☐	☐	☐
Beekeeping journals	☐	☐	☐	☐	☐
Internet, internet forums	☐	☐	☐	☐	☐

2/ Visited apiary

Location: postal code*: .... Municipality*: ....

Cadastral community: ....

GPS (6 decimals):

Latitude*:.. - Longitude*: .... (e.g.: 43.612454 -7.054878)

Sea level*:..........................

Which type of landuse can be found in a 3 km radius (approximate estimation)?

- ☐ Farmland: .... percent
- ☐ Meadows: .... percent
- ☐ Woods: .... percent
- ☐ Settlements: ....percent

Did you migrate the colonies of this apiary in the season 2015? ☐ yes ☐ no

IF yes; migratory movements 2015:

Crop (nectar, pollen, honeydew source)	Country/federal state	Date of movement to crop	Date of movement from crop

3/ Risk factors for the introduction of exotic animals

Have queens been imported to Austria for this company since the start of the year 2013?

☐ yes ☐ no

If yes, please state the country/countries of origin: ....

Have colonies, swarms or package bees been imported to Austria for this company since the start of the year 2013?

☐ yes ☐ no

If yes, please state the country/countries of origin: ....

4/ Changes of colony number in the season 2014/2015

a) Overwintering in this apiary

- Number of wintered colonies in autumn 2014*: ....

- Number of colonies after wintering in spring 2015*: ....

b) Colonies added to the apiary in the year 2015 till now*: ....(total)

- Thereof bought or taken from another apiary*: ....
  (if not from Austria, country of origin: ....)
- Thereof nucs*: ....
- Thereof natural swarms and artificial swarms (package bees)*:........................

c) Colonies removed from the apiary since the end of winter 2015*: ....(total)

- Thereof sold or moved to another apiary*: ....
- Thereof terminated/eliminated by merging with another colony*: ....
- Thereof died*:........................

d) Newly introduced queens in this apiary in the year 2015*: .... (total)

- Thereof bought*:.................. (if not from Austria, country of origin: ....)
- Thereof produced by the beekeeper*: ....

5/ Chemical varroa treatments in this apiary*

Please specify all treatments in the last 12 months as accurately as possible!

Product or active substance (including concentration)	Dosage per colony	Way of application	Date of treatment	Duration of treatment

6/ Biotechnical and other varroa treatments in this apiary *

Please specify all treatments in the last 12 months as accurately as possible!

Type of treatment	Conducted	Date of treatment				
**Drone brood removal**	☐ yes ☐ no					
**Heat treatment – whole colony**	☐ yes ☐ no<br>device......................					
**Heat treatment – brood combs**	☐ yes ☐ no<br>device......................					
**Total brood removal**	☐ yes ☐ no					
**Brood interruption**	☐ yes ☐ no					
**Trap comb method**	☐ yes ☐ no					

7/ Winter losses 2014 / 2015

*This chapter deals with the winter losses of **ALL colonies of the beekeeping** operation and not only of the sampled apiary. The questions are from the internet platform 'bienenstand.at' from the University of Graz, which is also a part of 'Zukunft Biene'. Please answer this part also if the beekeeper had already participated in the survey of the University of Graz.*

Did the beekeeper participate in the winter loss survey 2014/2015 of the University of Graz (Bienenstand.at)?* ☐ yes ☐ no

In which district are the apiaries located?*

☐ in one district:..

☐ in more than one district

Where is the main apiary located? Please state the name of the municipality and postal code.*

Municipality...; Postal code: ....

How many apiaries are part of the beekeeping operation?*.....................

Are all these apiaries within a distance of about 15 km of each other?*

☐ yes ☐ uncertain ☐ no

How many colonies were overwintered in the winter 2014/15?*.............

How many colonies were lost in the winter 2014/2015*

- Because of dead colonies or empty hives?.............
- because of unsolvable queen problems?....................

List of samples taken

Please fill in accordingly

**A) Description of the colony***	**ID of focus colony**											
	**1**	**2**	**3**	**4**	**5**	**6**	**7**	**8**	**9**	**10**	**11**	**12**
Queen emerged in the year…												
Productive colony (**WV**) or young colony (**JV**)												
Young colony: artificial swarm (**KS**) or nuc (**A**)												
Colony migrated in this season: yes (J) or no (N)												
Hive type (if different: brood super + honey supers)												
Number of supers												
Number of frames per super												
Colony size<br>(1: very weak; 2: normal; 3: very strong)												

PLEASE check all that apply:

**B.1) Observed symptoms before colony opening***	**1**	**2**	**3**	**4**	**5**	**6**	**7**	**8**	**9**	**10**	**11**	**12**
Dead bees in front of the hive	☐	☐	☐	☐	☐	☐	☐	☐	☐	☐	☐	☐
Bees unable to fly, crawlers in front of the hive	☐	☐	☐	☐	☐	☐	☐	☐	☐	☐	☐	☐
Fecal marks	☐	☐	☐	☐	☐	☐	☐	☐	☐	☐	☐	☐
Trembling bees	☐	☐	☐	☐	☐	☐	☐	☐	☐	☐	☐	☐
Black and shiny bees	☐	☐	☐	☐	☐	☐	☐	☐	☐	☐	☐	☐
Rejected bees	☐	☐	☐	☐	☐	☐	☐	☐	☐	☐	☐	☐
Thrown out brood	☐	☐	☐	☐	☐	☐	☐	☐	☐	☐	☐	☐
Crowded entrance	☐	☐	☐	☐	☐	☐	☐	☐	☐	☐	☐	☐
Other (set footnote & describe below)												

Please note extraordinary observations and symptoms ():
e.g. dead colonies in the apiary, use of back-opening hives,…

Abbreviation hive types:
Z....................Zander
ZF ....Zander flach
DN = EM ....Deutschnormal, Einheitsmaß
BW..................Breitwabe
D....................Dadant
If other hive type is used, define abbreviation here:

List of samples taken

Please check all that apply:

**B.2) Observed Symptoms in the colony***	**1**	**2**	**3**	**4**	**5**	**6**	**7**	**8**	**9**	**10**	**11**	**12**
Patchy brood pattern	☐	☐	☐	☐	☐	☐	☐	☐	☐	☐	☐	☐
Cell cappings punctured	☐	☐	☐	☐	☐	☐	☐	☐	☐	☐	☐	☐
Cell cappings concave / discolored	☐	☐	☐	☐	☐	☐	☐	☐	☐	☐	☐	☐
Slumped larvae	☐	☐	☐	☐	☐	☐	☐	☐	☐	☐	☐	☐
Discolored larvae / pupae	☐	☐	☐	☐	☐	☐	☐	☐	☐	☐	☐	☐
Dead larvae	☐	☐	☐	☐	☐	☐	☐	☐	☐	☐	☐	☐
Typical AFB smell	☐	☐	☐	☐	☐	☐	☐	☐	☐	☐	☐	☐
Glue-like larval remains	☐	☐	☐	☐	☐	☐	☐	☐	☐	☐	☐	☐
Scales	☐	☐	☐	☐	☐	☐	☐	☐	☐	☐	☐	☐
Sacbrood symptoms (dead larvae with saclike appearance, gondola-shaped' scales)	☐	☐	☐	☐	☐	☐	☐	☐	☐	☐	☐	☐
Chalkbrood mummies	☐	☐	☐	☐	☐	☐	☐	☐	☐	☐	☐	☐
Bees with deformed wings	☐	☐	☐	☐	☐	☐	☐	☐	☐	☐	☐	☐
Varroa mites on bees	☐	☐	☐	☐	☐	☐	☐	☐	☐	☐	☐	☐
Varroa mites embedded in cell cappings	☐	☐	☐	☐	☐	☐	☐	☐	☐	☐	☐	☐
Suspicious mites (besides Varroa mites)	☐	☐	☐	☐	☐	☐	☐	☐	☐	☐	☐	☐
Suspicious eggs / larvae / beetles	☐	☐	☐	☐	☐	☐	☐	☐	☐	☐	☐	☐
Other (describe below)												

**C) Suspicion of ***	**1**	**2**	**3**	**4**	**5**	**6**	**7**	**8**	**9**	**10**	**11**	**12**
Varroosis	☐	☐	☐	☐	☐	☐	☐	☐	☐	☐	☐	☐
American Foulbrood (if suspicion: sample mandatory!)	☐	☐	☐	☐	☐	☐	☐	☐	☐	☐	☐	☐
European Foulbrood (if suspicion: sample mandatory!)	☐	☐	☐	☐	☐	☐	☐	☐	☐	☐	☐	☐
Sacbrood	☐	☐	☐	☐	☐	☐	☐	☐	☐	☐	☐	☐
Chalkbrood	☐	☐	☐	☐	☐	☐	☐	☐	☐	☐	☐	☐
Nosemosis (if suspicion: sample mandatory!)	☐	☐	☐	☐	☐	☐	☐	☐	☐	☐	☐	☐
Chronic Bee Paralysis Virus (if suspicion: sample mandatory!)	☐	☐	☐	☐	☐	☐	☐	☐	☐	☐	☐	☐
Deformed Wing Virus (if suspicion: sample mandatory!)	☐	☐	☐	☐	☐	☐	☐	☐	☐	☐	☐	☐
Small hive beetle (if suspicion: sample mandatory!)	☐	☐	☐	☐	☐	☐	☐	☐	☐	☐	☐	☐
Tropilaelaps mite (if suspicion: sample mandatory!)	☐	☐	☐	☐	☐	☐	☐	☐	☐	☐	☐	☐
Other (describe below)												
**Dead colony (only visit 2 or 3)**	☐	☐	☐	☐	☐	☐	☐	☐	☐	☐	☐	☐

List of samples taken

SAMPLING*	ID of focus colony						
	1	2	3	4	5	6	7
**Mandatory samples – note sample number**							
Bee sample Varroa<br>Large cup<br>**Visits 1 and 2**							
Bee sample Posthoc<br>Small cup<br>**All visits**							
Beebread sample<br>**All visits**							
**Samples taken, when suspicion for disease – stick label in correct place**							
Bees –<br>Sampled outside the colony							
Bees –<br>Sampled inside the colony							
Suspicious brood comb							
Suspicious mite							
Suspicious beetle/ beetle larvae							
Other (describe below)							
