# Supplementary material for: Health status of honey bee colonies (Apis mellifera) and disease-related risk factors for colony losses in Austria
Source: PLoS One. 2019 Jul 9;14(7):e0219293. doi: 10.1371/journal.pone.0219293 (PMC6615611; doi:10.1371/journal.pone.0219293)
Supplement: S4 Text — (PDF) [file pone.0219293.s004.pdf]

S5 Text. Effect of missing cases on modelling of colony losses.

Summer visit

For the summer visit, varroa mite samples from 33 colonies were missing (missing cases), partly due to the weakness of the colony (29) and partly due to logistic problems (4 colonies). These colonies were therefore not included into the multivariate models. The missing out of weak colonies in the model may alter the modelling result, as weak colonies could be especially endangered to suffer a colony loss. However, the missing cases from summer did not differ in the probability of summer loss or winter loss compared to the rest of the sample (see table below). This may be connected with the reasons for the weakness of the colonies. From the 29 weak colonies, 24 colonies were not sampled in summer because they were nucleuses produced in that year and were spared by the bee inspectors to help them increasing their colony size undisturbed. These colonies were small because of their development status and not because of a problem of the colony. Another colony was not sampled due to a recent requeening event. Only four colonies from the total of 29 weak colonies were too small for sampling without any reason connected with the particular beekeeping practice.

Table a. Colony losses in the group with missing data of the varroa mite infestation rate in September (missing cases) and in the group without missing data (rest of the sample).

Visit	Time of loss	N° missing cases/ total sample	Percentage of loss (95%CI)		Significance level
			Missing cases	Rest of sample	
Summer visit	summer	33 / 1596	0 %	2.7 % (2.0-3.6 %)	P = 0.984
	winter	33 / 1554	12.1 % (3.9-26.0 %)	8.3 % (7.0-9.8 %)	P = 0.444
Autumn visit	winter	23 / 1554	30.4 % (14.4-50.6 %)	8.1 % (6.8-9.5 %)	P < 0.001

Autumn visit

For the autumn visit, varroa mite samples from 23 colonies were missing (missing cases) due to the weakness of the colony. These 23 colonies had a three times increased probability to die over the winter in comparison to the rest of the sample (see table). Therefore we checked with univariate modelling, if the missing out of these cases may alter the modelled relationship between colony strength and winter loss (see figure a, table b). The results did not show a difference between the models with or without these 23 cases. Figure a shows that the 95%Confidence Intervals of the group "weak" between the two models overlap in large parts. When comparing the model estimates, again the odds of the different variable levels differed not significantly between the two models (Table b, range of the 95%CI overlaps).

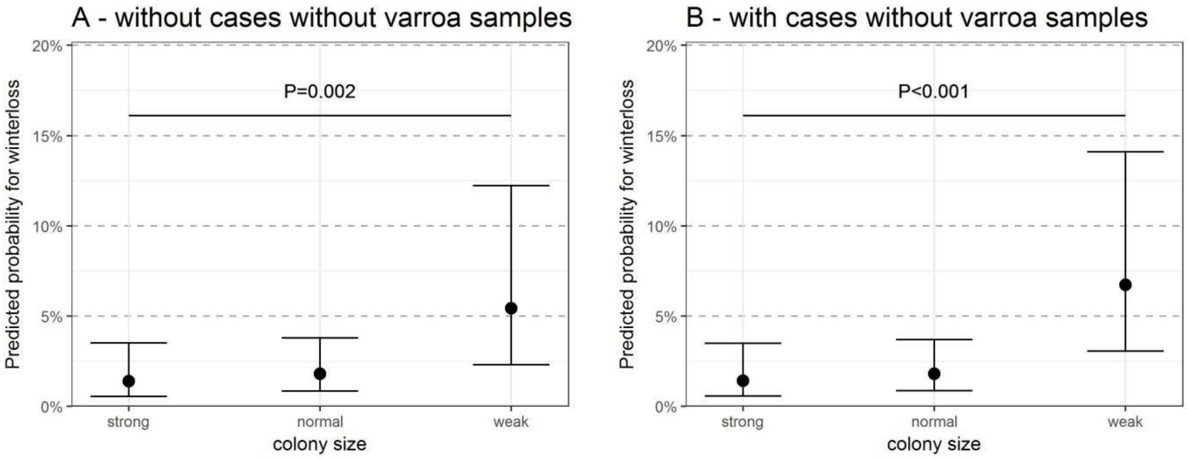

Fig a. Probability for winter loss as predicted by two univariate GLMMs (binomial distribution, random factor: apiary) differing in the size of the dataset. Model of (A) excluded the 23 colonies, from which the varroa mite samples of September were missing (n=1498 colonies). Model of (B) included that 23 cases (n=1521colonies). For both models: N = 188 apiaries.

Table b. Relationship between colony strength in autumn and winter loss in the following winter. Model was calculated with two datasets. 'without missing cases' excluded 23 colonies, from which the varroa mite samples of September were missing (n=1498 colonies), 'with missing cases' included all 1521 colonies, of which colony strength in September was reported. GLMM with binomial distribution, random factor 'apiary'. n = 1521 colonies; N = 188 apiaries.

Model	Variable	Levels	Estimate (SE)	Odds (95% CI)	Z value	P
without missing cases	Intercept		-4.25 (0.48)	0.01 (0.01-0.04)	-8,85	<0.001
	Colony strength autumn reference level: strong colony	Normal	0.26 (0.36)	1.29 (0.64-2.6)	0,72	0.470
		Weak	1.4 (0.45)	4.04 (1.67-9.74)	3,11	0.002
with missing cases	Intercept		-4.24 (0.47)	0.01 (0.01-0.04)	-9.02	<0.001
	Colony strength autumn reference level: strong colony	Normal	0.24 (0.36)	1.28 (0.63-2.56)	0.67	0.494
		Weak	1.61 (0.43)	5.01 (2.14-11.73)	3.74	<0.001
