# Supplementary material for: Health status of honey bee colonies (Apis mellifera) and disease-related risk factors for colony losses in Austria
Source: PLoS One. 2019 Jul 9;14(7):e0219293. doi: 10.1371/journal.pone.0219293 (PMC6615611; doi:10.1371/journal.pone.0219293)
Supplement: S1 Table — (PDF) [file pone.0219293.s006.pdf]

S6 Table. Primers and PCR-parameters for the tests for *Nosema apis*, *Nosema ceranae* and CBPV.

**Agent**	**Primers (5´-3´)**	**Amplifikation product [bp]**	**Authors**
N. apis	GGG GGC ATG TCT TTG ACG TAC TAT GTA<br>GGG GGG CGT TTA AAA TGT GAA ACA ACT ATG	321	Martín-Hernández et al. (2007)
N. ceranae	CGG CGA CGA TGT GAT ATG AAA ATA TTA A<br>CCC GGT CAT TCT CAA ACA AAA AAC CG	218	Martín-Hernández et al. (2007)
CBPV	TCA GAC ACC GAA TCT GAT TAT TG<br>ACT ACT AGA AAC TCG TCG CTT CG	570	Blanchard et al. (2008)

Martín-Hernández R, Meana A, Prieto L, Salvador AM, Garrido-Bailón E, Higes M. Outcome of Colonization of Apis mellifera by Nosema ceranae. Appl Environ Microbiol. 2007;73(20):6331-8.

Blanchard P, Olivier V, Iscache A-L, Celle O, Schurr F, Lallemand P, et al. Improvement of RT-PCR detection of chronic bee paralysis virus (CBPV) required by the description of genomic variability in French CBPV isolates. J Invertebr Pathol. 2008;97(2):182-5.
