# Supplementary material for: Health status of honey bee colonies (Apis mellifera) and disease-related risk factors for colony losses in Austria
Source: PLoS One. 2019 Jul 9;14(7):e0219293. doi: 10.1371/journal.pone.0219293 (PMC6615611; doi:10.1371/journal.pone.0219293)
Supplement: S2 Table — Correlation between clinical prevalence of six diseases in the observed 1596 colonies and survival of the colonies in summer 2015 (between July 2015 –September 2015) and the winter season 2015/16, respectively. The percentage of dead colonies per group (= row) is given in brackets. Each variable is tested with a Chi2-Test or a Fisher’s Exact Test (FET), respectively. Diseases without positive cases were not tested and are therefore not shown. Significant results are shaded in gray. (PDF) [file pone.0219293.s007.pdf]

S7 Table. Clinical prevalence of six bee diseases and colony losses. Correlation between clinical prevalence of six diseases in the observed 1596 colonies and survival of the colonies in summer 2015 (between July 2015 – September 2015) and the winter season 2015/16, respectively. The percentage of dead colonies per group (=row) is given in brackets. Each variable is tested with a Chi^2-Test or a Fisher's Exact Test (FET), respectively. Diseases without positive cases were not tested and are therefore not shown. Significant results are shaded in gray.

Visit	Observed disease	Symptoms	Summer losses			Winter losses		
			N° colonies alive autumn visit	N° colonies dead in summer	Effect on summer losses?	N° colonies alive spring visit	N° colonies dead in winter	Effect on winter losses?
First visit summer 2015 N=1598*	AFB	Negative	1553	39 (2.4 %)	P < 0.001, FET	1422	131 (8.4 %)	---
		Positive	0	3 (100.0 %)		0	0	
	CBPV	Negative	1552	42 (2.6 %)	P = 1.000, FET	1421	131 (8.4 %)	P = 1.000, FET
		Positive	1	0 (0.0 %)		1	0 (0.0 %)	
	Chalkbrood	Negative	1495	42 (2.7 %)	P = 0.441 FET	1368	127 (8.5 %)	Chi^2 = 0.036, df = 1, P = 0.8501
		Positive	58	0 (0.0 %)		54	4 (6.9 %)	
	Nosemosis	Negative	1550	42 (2.6 %)	P = 1.000, FET	1420	130 (8.4 %)	P = 0.232, FET
		Positive	3	0 (0.0 %)		2	1 (33.3%)	
	Sacbrood	Negative	1534	41 (2.6 %)	P = 0.416 FET	1405	129 (8.4 %)	P = 0.695, FET
		Positive	19	1 (5.0 %)		17	2 (10.5 %)	
	Varroosis/ DWV	Negative	1479	33 (2.2 %)	P < 0.001 FET	1363	116 (7.8 %)	Chi^2 = 12.528, df = 1, P < 0.001
		Positive	74	9 (10.8 %)		59	15 (20.3 %)	
Second visit autumn 2015 N=1554	AFB	Negative				1423	131 (8.4 %)	---
		Positive				0	0	
	CBPV	Negative				1421	131 (8.4 %)	P = 1.000, FET
		Positive				2	0 (0.0 %)	
	Chalkbrood	Negative				1412	131 (8.5 %)	P = 1.000, FET
		Positive				11	0 (0.0 %)	
	Nosemosis	Negative				1423	131 (8.4 %)	---
		Positive				0	0	
	Sacbrood	Negative				1421	130 (8.4 %)	P = 0.232, FET
		Positive				2	1 (33.3 %)	
	Varroosis /DWV	Negative				1360	113 (7.7 %)	Chi^2 = 19.216, df = 1, P < 0.001
		Positive				63	18 (22.2 %)	

*one missing value, because colony was not inspected, it survived the winter 2015/16
