# Supplementary material for: Health status of honey bee colonies (Apis mellifera) and disease-related risk factors for colony losses in Austria
Source: PLoS One. 2019 Jul 9;14(7):e0219293. doi: 10.1371/journal.pone.0219293 (PMC6615611; doi:10.1371/journal.pone.0219293)
Supplement: S3 Table — The percentage of dead colonies per group (= row) is given in brackets. Each variable is tested with a Chi2-Test or a Fisher’s Exact Test (FET), respectively. Number of missing values for each Variable and testing periods are given. n = 1569 colonies. (PDF) [file pone.0219293.s008.pdf]

S8 Table. Categorical colony characteristics related with survival in summer 2015 (summer losses) and winter 2015/16 (winter losses), respectively. The percentage of dead colonies per group (=row) is given in brackets. Each variable is tested with a Chi²-Test or a Fisher's Exact Test (FET), respectively. Number of missing values for each Variable and testing periods are given. n = 1569 colonies.

			Summer losses			Winter losses		
**Variable**	**Missing values**	**Levels**	**N° colonies alive autumn visit**	**N° colonies dead in summer**	**Effect on summer losses?**	**N° colonies alive spring visit**	**N° colonies dead in winter**	**Effect on winter losses?**
Type of the colony in spring	Summer: 41; Winter: 40	Nuc/swarm	307	5 (1.6 %)	Chi² = 1.161, df = 1, P = 0.281	292	15 (4.9 %)	Chi² = .054, df = 1, P = 0.025
		Productive colony	1207	36 (2.9 %)		1098	109 (9.0 %)	
Migrating events in spring	Summer: 57; Winter: 57	Colony migrated	199	5 (2.5 %)	Chi² = 0.000, df = 1, P = 0.975	189	10 (5.0 %)	Chi² = 2.147, df = 1, P = 0.143
		Colony not migrated	1298	37 (2.8 %)		1190	108 (8.3 %)	
Colony size summer visit	Summer: 65; Winter: 65	Weak	185	19 (9.3 %)	Chi² = 38.192, df = 2, P < 0.001	166	19 (10.3 %)	Chi² = 1.595, df = 2, P = 0.450
		Normal	848	14 (1.6 %)		782	66 (7.8 %)	
		strong	456	9 (1.9 %)		414	42 (9.2 %)	
Colony size autumn visit	Winter: 33	Weak				147	35 (19.2 %)	Chi² = 30.389, df = 2, P < 0.001
		Normal				955	75 (7.3 %)	
		strong				289	20 (6.5 %)	
Queen age at time of visit	Summer: 123; Winter: 121	2 years and more	261	5 (1.9 %)	Chi² = 2.355, df = 2, P = 0.308	168	26 (13.4 %)	Chi² = 14.99, df = 2, P < 0.001
		1 year	615	19 (3.0 %)		472	50 (9.6 %)	
		0 years	563	10 (1.7 %)		677	40 (5.6 %)	
Queen problems	Summer: 106; Winter: 97	Queen problem	13	4 (23.5 %)	FET < 0.001	21	3 (12.5 %)	FET: P = 0.439
		Normal queen	1439	34 (2.4 %)		1317	116 (8.1 %)	
Winter food	Winter: 0	Sugar				645	59 (8.4 %)	Chi² = 0.954, df = 2, P = 0.621
		Commercial mixture				683	66 (8.8 %)	
		Sugar + commercial				95	6 (5.9 %)	
Highest beekeeper education	Summer: 0; Winter: 0	No education	176	1 (0.6 %)	Chi² = 5.679, df = 3, P = 0.128	160	16 (9.1 %)	Chi² = 2.506, df = 3, P = 0.474
		Beginners' course	146	2 (1.4 %)		132	14 (9.6 %)	
		advanced courses	610	22 (3.5 %)		567	43 (7.0 %)	
		Professional training	622	17 (2.7 %)		564	58 (9.3 %)	
Organic beekeeping	Summer: 0; Winter: 0	Conventional	1264	34 (2.6 %)	Chi² = 0.000, df = 1, P = 1.000	1160	104 (8.2 %)	Chi² = 0.232, df = 1, P = 0.630
		Organic	290	8 (2.7 %)		263	27 (9.3 %)	
