# Supplementary material for: Health status of honey bee colonies (Apis mellifera) and disease-related risk factors for colony losses in Austria
Source: PLoS One. 2019 Jul 9;14(7):e0219293. doi: 10.1371/journal.pone.0219293 (PMC6615611; doi:10.1371/journal.pone.0219293)
Supplement: S4 Table — For each variable the number of cases (N), the median and the first (Q1) and third quartile (Q3) are given. Differences between the groups of alive and dead colonies were tested with a Wilcoxon Rank Sum Test (significant differences highlighted in gray). cols = colonies, yrs = years. n = 1569 colonies. (PDF) [file pone.0219293.s009.pdf]

**S9 Table. Metric colony characteristics related with survival in summer 2015 (summer losses) and winter 2015/16 (winter losses), respectively.** For each variable the number of cases (N), the median and the first (Q1) and third quartile (Q3) are given. Differences between the groups of alive and dead colonies were tested with a Wilcoxon Rank Sum Test (significant differences highlighted in gray). cols = colonies, yrs = years. n = 1569 colonies.

| Season of loss | Variable | Colonies alive after the season | | Colonies dead after the season | | statistics |
|---|---|---|---|---|---|---|
| | | N | Median (Q1-Q3) | N | Median (Q1-Q3) | |
| Summer losses | Varroa infestation level summer | 1521 | 0.3 % (0.0-0.8 %) | 42 | 1.3 % (0.0-2.8 %) | W = 20754, P < 0.001 |
| | Experience as a beekeeper | 1554 | 26.0 yrs (12.0-35.0 yrs) | 42 | 31.0 yrs (10.0-48.0 yrs) | W = 29162, P = 0.239 |
| | Company size | 1544 | 33.0 (17.0-70.0 cols) | 42 | 20 cols (9.0-54.0 cols) | W = 39614, P = 0.014 |
| | Apiary size | 1544 | 15.0 cols (10.0-20.0 cols) | 42 | 9 cols (8.0-17.0 cols) | W = 40349, P = 0.009 |
| Winter losses | Varroa infestation level summer | 1394 | 0.1 % (0.0-0.8 %) | 127 | 0.6 % (0.0-2.3 %) | W = 111430, P < 0.001 |
| | Varroa infestation level autumn | 1407 | 0.5 % (0.0-1.8 %) | 124 | 3.5 % (0.8%-10.4 %) | W = 129220, P < 0.001 |
| | Experience as a beekeeper | 1423 | 27.0 yrs (13.0-35.0 yrs) | 131 | 20.0 yrs (9.0-31.0 yrs) | W = 107240, P = 0.004 |
| | Company size | 1415 | 33.0 cols (17.0-75.0 cols) | 129 | 37.0 cols (22.0-55.0 cols) | W = 93348, P = 0.668 |
| | Apiary size | 1423 | 15.0 cols (10.0-21.0 cols) | 131 | 15.0 cols (8.5-20.0 cols) | W = 101430, P = 0.094 |
| | Honey harvest per colony | 1392 | 28.0 kg (20.0-40.0 kg) | 131 | 27.0 kg (20.0-35.0 kg) | W = 93283, P = 0.661 |
