# Supplementary material for: Health status of honey bee colonies (Apis mellifera) and disease-related risk factors for colony losses in Austria
Source: PLoS One. 2019 Jul 9;14(7):e0219293. doi: 10.1371/journal.pone.0219293 (PMC6615611; doi:10.1371/journal.pone.0219293)
Supplement: S5 Table — The percentage of weak colonies per group (= row) is given in brackets. Each variable is tested with a Chi2-Test or a Fisher’s Exact Test (FET), respectively. Significant results are shaded in gray. (PDF) [file pone.0219293.s010.pdf]

S10 Table. Correlation between small size of the colonies and colony characteristics, diseases. The percentage of weak colonies per group (=row) is given in brackets. Each variable is tested with a Chi²-Test or a Fisher's Exact Test (FET), respectively. Significant results are shaded in gray.

Variable	Levels	Summer visit			Autumn visit		
		N° colonies rated strong or normal	N° (percentage) colonies rated very small	Statistics	N° colonies rated strong or normal	N° (percentage) colonies rated very weak	Statistics
Queen problems	Normal queen	1234	178 (13 %)	FET: $P<0.001$	1236	164 (12%)	FET: $P=0.005$
	Queen problem	7	10 (59 %)		16	8 (33%)	
Queen age at time of visit	2 years and more	239	21 (8 %)	Chi² = 36.044, df = 2, $P < 0.001$	171	20 (10%)	Chi² = 9.525, df = 2, $P = 0.009$
	1 year	556	52 (9 %)		466	44 (9%)	
	0 years	439	105 (19 %)		599	100 (14%)	
Type of the colony in spring	Nuc/swarm	223	70 (24 %)	Chi² = 44.921, df = 1, $P < 0.001$	245	52 (18 %)	Chi² = 9.459, df = 1, $P = 0.002$
	Productive colony	1060	109 (9 %)		1059	128 (11 %)	
Varroa level at time of visit	0.00 %	624	104 (14 %)	Chi² = 6.537, df = 2, $P = 0.038$	408	52 (11 %)	Chi² = 7.488, df = 2, $P = 0.024$
	0.01 %-3.00 %	572	64 (10 %)		680	67 (9 %)	
	>3.00 %	122	13 (10 %)		248	43 (15 %)	
AFB	Negative	1326	202 (13%)	FET: $P = 0.048$	1339	182 (12 %)	---
	Positive	1	2 (67%)		0	0 (0%)	
CBPV	Negative	1326	204 (13%)	---	1337	182 (12 %)	FET: $P = 1.000$
	Positive	1	0 (0%)		2	0 (0 %)	
Chalkbrood	Negative	1276	197 (13%)	Chi² = 0.008, df = 1, $P = 0.928$	1328	182 (12 %)	FET: $P = 0.380$
	Positive	51	7 (12%)		11	0 (0%)	
Nosemosis	Negative	1324	204 (13%)	FET: $P = 1.000$	1339	182 (12 %)	---
	Positive	3	0 (0%)		0	0 (0%)	
Sacbrood	Negative	1312	199 (13%)	FET: $P = 0.172$	1338	180 (12%)	FET: $P = 0.039$
	Positive	15	5 (25%)		1	2 (67%)	
Varroosis/ DWV	Negative	1269	184 (13%)	Chi² = 9.70, df = 1, $P = 0.002$	1276	164 (11 %)	Chi² = 7.546, df = 1, $P = 0.006$
	Positive	58	20 (26%)		63	18 (22 %)	
