# Supplementary material for: Health status of honey bee colonies (Apis mellifera) and disease-related risk factors for colony losses in Austria
Source: PLoS One. 2019 Jul 9;14(7):e0219293. doi: 10.1371/journal.pone.0219293 (PMC6615611; doi:10.1371/journal.pone.0219293)
Supplement: S6 Table — The percentage of weak colonies per group (= row) is given in brackets. Each variable is tested with a Chi2-Test or a Fisher’s Exact Test (FET), respectively. Significant results are shaded in gray. (PDF) [file pone.0219293.s011.pdf]

S11 Table – part 1. Correlation between small size of the colonies and disease symptoms. The percentage of weak colonies per group (=row) is given in brackets. Each variable is tested with a Chi²-Test or a Fisher's Exact Test (FET), respectively. Significant results are shaded in gray.

Variable	Levels	Summer visit			Autumn visit		
		N° colonies rated strong or normal	N° (percentage) colonies rated very small	Statistics	N° colonies rated strong or normal	N° (percentage) colonies rated very weak	Statistics
Dead bees in front of the hive	Negative	1301	199 (13 %)	FET: P = 0.594	1326	182 (12 %)	FET: P = 0.3869
	Positive	26	5(16 %)		13	0 (0 %)	
Bees unable to fly, crawlers in front of the hive	Negative	1307	203 (13 %)	FET: P = 0.344	1332	179 (12 %)	FET: P = 0.107
	Positive	20	1 (5 %)		7	3 (30 %)	
Fecal marks	Negative	1326	203 (13 %)	FET: P = 0.249	1336	182 (12 %)	FET: P = 1.000
	Positive	1	1 (50 %)		3	0 (0 %)	
Trembling bees	Negative	1325	204 (13 %)	FET: P = 1.000	1338	182 (12 %)	FET: P = 1.000
	Positive	2	0 (0 %)		1	0 (0 %)	
Black and shiny bees	Negative	1288	200 (13 %)	Chi² = 0.313, df = 1, P = 0.576	1274	169 (12 %)	Chi² = 1.286, df = 1, P = 0.257
	Positive	39	4 (9 %)		65	13 (17 %)	
Rejected bees	Negative	1320	204 (13 %)	FET: P = 0.604	1339	182 (13 %)	---
	Positive	7	0 (0 %)		0	0	
Thrown out brood	Negative	1327	204 (13 %)	---	1338	179 (12 %)	FET: P = 0.006
	Positive	0	0		1	3 (75 %)	
Crowded entrance	Negative	1327	204 (13 %)	---	1339	182 (13 %)	---
	Positive	0	0		0	0	
Patchy brood pattern	Negative	1217	170 (12 %)	Chi² = 13.596, df = 1, P < 0.001	1257	160 (11 %)	Chi² = 8.035, df = 1, P = 0.005
	Positive	110	34 (24 %)		82	22 (21 %)	
Cell cappings punctured	Negative	1313	192 (13 %)	FET: P < 0.001	1336	179 (12 %)	FET: P = 0.026
	Positive	14	12 (46 %)		3	3 (50 %)	

S11 Table – part 2. Correlation between small size of the colonies and disease symptoms. The percentage of weak colonies per group (=row) is given in brackets. Each variable is tested with a Chi²-Test or a Fisher's Exact Test (FET), respectively. Significant results are shaded in gray.

Variable	Levels	Summer visit			Autumn visit		
		N° colonies rated strong or normal	N° (percentage) colonies rated very small	Statistics	N° colonies rated strong or normal	N° (percentage) colonies rated very weak	Statistics
Cell cappings concave / discolored	Negative	1318	198 (13 %)	FET: P < 0.01	1338	182 (12 %)	FET: P = 1.000
	Positive	9	6 (40 %)		1	0 (50 %)	
Slumped larvae	Negative	1324	198 (13 %)	FET: P < 0.001	1337	182 (12 %)	FET: P = 1.000
	Positive	3	6 (67 %)		2	0 (0 %)	
Discolored larvae / pupae	Negative	1325	199 (13 %)	FET: P < 0.001	1338	182 (12 %)	FET: P = 1.000
	Positive	2	5 (71 %)		1	0 (0 %)	
Dead larvae	Negative	1316	192 (13 %)	FET: P < 0.001	1330	176 (12 %)	FET: P = 0.005
	Positive	11	12 (52 %)		9	6 (40 %)	
Glue-like larval remains	Negative	1326	202 (13 %)	FET: P = 0.048	1339	182 (12 %)	---
	Positive[a]	1[a]	2[a] (67 %)		0	0	
Scales	Negative	1327	203 (13 %)	FET: P = 0.133	1339	182 (12 %)	---
	Positive[a]	0	1[a] (100 %)		0	0	
Bees with deformed wings	Negative	1307	193 (13 %)	FET: P = 0.001	1310	169 (11 %)	Chi² = 12.986, df = 1, P < 0.001
	Positive	20	11 (35 %)		29	13 (31 %)	
Varroa mites on bees	Negative	1290	191 (13 %)	Chi² = 6.101, df = 1, P = 0.014	1294	173 (12 %)	Chi² = 0.757, df = 1, P = 0.384
	Positive	37	13 (26 %)		45	9 (17 %)	
Varroa mites embed-ded in cell cappings	Negative	1314	202 (13 %)	FET: P = 1.000	1335	179 (12 %)	FET: P = 0.041
	Positive	13	2 (13 %)		4	3 (43 %)	

[a]: confirmed cases of American Foulbrood, all colonies died
