# Supplementary material for: Health status of honey bee colonies (Apis mellifera) and disease-related risk factors for colony losses in Austria
Source: PLoS One. 2019 Jul 9;14(7):e0219293. doi: 10.1371/journal.pone.0219293 (PMC6615611; doi:10.1371/journal.pone.0219293)
Supplement: S7 Table — 95%CI is given in brackets. Prevalence was evaluated by checking the bee colonies for clinical signs (Table 1). Cases with signs of American Foulbrood, CBPV and Nosemosis were confirmed by using standard laboratory tests. Sampling was done in summer 2015 (July, August), autumn 2015 (September, October) and spring 2016 (March-May). (PDF) [file pone.0219293.s012.pdf]

S12 Table. Prevalence of bee diseases and pests in the study colonies. 95%CI is given in brackets. Prevalence was evaluated by checking the bee colonies for clinical signs (Table 1). Cases with signs of American Foulbrood, Chronic Bee Paralysis Virus (CBPV) and Nosemosis were confirmed by using standard laboratory tests. Sampling was done in summer 2015 (July, August), autumn 2015 (September, October) and spring 2016 (March-May).

		Apiary level			Colony level		
		Summer n=189	Autumn n=188	Spring n=183	Summer n=1595	Autumn n=1544	Spring n=1401
notifiable disease	American Foulbrood	0.5 % (0.1-1.9)	0.0 % (0.0-0.0)	0.6 % (0.1-1.9)	0.2 % (0.1-0.4)	0.0 % (0.0-0.0)	0.1 % (0.0-0.3)
	Small Hive Beetle	0.0 % (0.0-0.0)	0.0 % (0.0-0.0)	0.0 % (0.0-0.0)	0.0 % (0.0-0.0)	0.0 % (0.0-0.0)	0.0 % (0.0-0.0)
	Tropilaelaps mite	0.0 % (0.0-0.0)	0.0 % (0.0-0.0)	0.0 % (0.0-0.0)	0.0 % (0.0-0.0)	0.0 % (0.0-0.0)	0.0 % (0.0-0.0)
	Varroosis	21.2 % (15.7-27.4)	21.8 % (16.3-28.1)	7.7 % (4.4-12.1)	5.2 % (4.2-6.4)	5.2 % (4.2-6.4)	1.6 % (1.0-2.3)
not notifiable disease	CBPV	0.5 % (0.1-1.9)	1.1 % (0.3-2.8)	0.0 % (0.0-0.0)	0.1 % (0.0-0.2)	0.1 % (0.0-0.3)	0.0 % (0.0-0.0)
	European Foulbrood	0.0 % (0.0-0.0)	0.0 % (0.0-0.0)	0.0 % (0.0-0.0)	0.0 % (0.0-0.0)	0.0 % (0.0-0.0)	0.0 % (0.0-0.0)
	Chalkbrood	12.7 % (8.5-18.0)	4.3 % (2.0-7.8)	8.7 % (5.2-13.4)	3.6 % (2.8-4.6)	0.7 % (0.4-1.2)	2.2 % (1.5-3.1)
	Nosemosis	1.1 % (0.3-2.7)	0.0 % (0.0-0.0)	1.1 % (0.3-2.8)	0.2 % (0.1-0.4)	0.0 % (0.0-0.0)	0.4 % (0.2-0.7)
	Sacbrood	6.9 % (3.8-11.1)	1.6 % (0.4-4.1)	1.6 % (0.4-4.2)	1.3 % (0.8-1.9)	0.2 % (0.1-0.5)	0.2 % (0.1-0.6)
