# Supplementary material for: Health status of honey bee colonies (Apis mellifera) and disease-related risk factors for colony losses in Austria
Source: PLoS One. 2019 Jul 9;14(7):e0219293. doi: 10.1371/journal.pone.0219293 (PMC6615611; doi:10.1371/journal.pone.0219293)
Supplement: S8 Table — The losses are calculated for two different definitions of winter loss: (1) dead colonies and (2) dead colonies and living colonies with unsolvable queen problems in spring. 95%CI = 95% Confidence Interval. (PDF) [file pone.0219293.s013.pdf]

S13 Table. Winter losses of study colonies, participating apiaries (N=188) and participating operations (N=188). The losses are calculated for two different definitions of winter loss: (1) dead colonies and (2) dead colonies and living colonies with unsolvable queen problems in spring. 95%CI = 95 % Confidence Interval.

Sample group	N° colonies in autumn	N° dead colonies in spring	N° queenless colonies in spring	Winter loss – only dead colonies [Percentage (95%CI)]	Winter loss – dead + queenless colonies [Percentage (95%CI)]
Beekeeping operation	12 540	659	421	5.3 % (4.1-6.7 %)	8.6 % (7.5-9.9 %)
Sample apiary	2 810	211	74	7.5 % (5.8-9.7 %)	10.1 % (8.3-12.4 %)
Sample colonies	1 554	131	32	8.4 % (7.1-9.9 %)	10.5 % (9.0-12.1 %)
